# RGN as a prognostic biomarker with immune infiltration and ceRNA in lung squamous cell carcinoma

**DOI:** 10.1038/s41598-023-32217-z

**Published:** 2023-05-09

**Authors:** Yang Liao, Wen Cheng, Ruiyu Mou, Xiaojiang Li, Yingjie Jia

**Affiliations:** 1 Department of Oncology, First Teaching Hospital of Tianjin University of Traditional Chinese Medicine, Tianjin, 300008 China; 2 National Clinical Research Center for Chinese Medicine Acupuncture and Moxibustion, Tianjin, 300008 China

**Keywords:** Cancer, Tumour biomarkers

## Abstract

Regucalcin (RGN) is a potent inhibitory protein of calcium signaling and expresses in various tissues. However, the role of RGN in the tumor immunological microenvironment in lung squamous cell carcinoma (LUSC) remains unclear. This study identified the expression of RGN from public databases and immunohistochemistry with clinical specimen. The association between RGN and the tumor immune microenvironment (TIME) was investigated in LUSC by ESTIMATE and CIBERSORT algorithms. Similarly, the Tumor IMmune Estimation Resource (TIMER) database was used to identify the correlation between RGN and immune cells. The ceRNA network was established based on the data obtained from public databases. Finally, prediction of drug response to chemotherapy and immunotherapy was performed to evaluate clinical significance. This study found that RGN expression was significantly downregulated in tumor tissues and closely related to clinical factors and prognosis of LUSC patients. Differentially expressed genes (DEGs) grouped by the expression of RGN were mostly involved in immunobiological processes such as humoral immune response and leukocyte mediated immunity. RGN and its related miRNA (has-miR-203a-3p) and lncRNAs (ZNF876P and PSMG3-AS1) constructed the novel prognosis-related ceRNA network. Plasma cells, T cells CD4 memory resting, Macrophages M0, Macrophages M1, Mast cells resting, Mast cells activated and Neutrophils showed significantly different levels of infiltration between high and low RGN expression groups. The TIMER database showed that RGN expression was positively correlated with certain immune infiltrating cells. High RGN expression group showed a higher TIDE score, a higher dysfunction score and a lower MSI score, presenting a possible lower efficacy after accepting the immunotherapy than low RGN expression group. RGN expression was closely associated with prognosis of LUSC patients and played an important role in tumor microenvironment. This suggests that RGN could be a promising biomarker for assessing immunotherapy efficacy and prognosis.

## Introduction

As a leading cause of cancer-related mortality, lung cancer is among the deadliest malignancies globally, and nearly 1.8 million people are diagnosed with lung cancer every year^1^. Small cell lung cancer (SCLC) and non-small cell lung cancer (NSCLC) are two types of lung cancer. Lung squamous cell carcinoma (LUSC) accounting for approximately 40% of all lung cancer is a highly aggressive subtype of NSCLC, and its diverse histological morphology is associated with poor clinical prognosis^2^. The landscape of LUSC treatments has gradually evolved from chemotherapy surgery to immunotherapies and combined treatments in different stage of LUSC patients. Importantly, several molecular targets such as programmed death-ligand 1 (PD-L1) was identified and demonstrated high sensitivity and response rates for immunotherapies^3,4^. Notwithstanding, therapeutic resistance and tumor recurrence appear to be major therapeutic challenges in the treatment of LUSC^5^. Hence, novel essential biomarkers are urgently needed to develop and change treatment strategies in the era of precision medicine.

The tumor immune microenvironment (TIME), which contains tumor, immune, stromal, and extracellular components, strongly supports the behavior of the tumor in terms of progression and metastasis^6^. As a critical determinant of cancer evolution and outcome, TIME tends to associated with different clinical outcomes based on different immune infiltration levels. A recent study indicated that the TIME of LUSC is characterized by immunogenic and heterogenous, with predominant infiltration of activated CD8+ T cells^7^. For Chinese LUSC patients, low tumor mutation burden (TMB) and high CD8+ tumor infiltrating lymphocytes (TILs) density were independently associated with longer disease-free survival (DFS), which presented remarkable correlation between the TIME and LUSC^8^.

The regucalcin (RGN) gene, which is located on the X chromosome in humans, encodes a potent inhibitory protein of calcium signaling and expresses in various types of cells and tissues^9,10^. As a senescence marker protein closely correlated with osteoporosis, the expression of RGN is regulated by the aging process^11,12^. Through maintaining intracellular Ca^2+^ homeostasis and suppressing signal transduction in diverse cells, regucalcin plays a huge role in regulating cellular functions and physiological processes. Furthermore, a study showed that the induction of RGN expression in Min6 cells was effective in suppressing lipopolysaccharide (LPS)-induced inflammatory cytotoxicity in the TIME^13^. However, the role of RGN and its specific mechanisms in the crafting of the tumor immunological microenvironment in LUSC remain unclear.

In this study, we evaluated the relationship between RGN and prognosis of LUSC patients based on the data from TCGA and GEO databases. Notably, the association between RGN expression and the TIME was investigated in LUSC by bioinformatics techniques including ESTIMATE and CIBERSORT algorithms. All results provide novel insights into the functional role and prognostic value of RGN in LUSC, and reveal its potential mechanistic basis related to immune infiltration.

## Materials and methods

### Data and sources

The Cancer Genome Atlas (TCGA: https://cancergenome.nih.gov/) was used to obtain the expression profile of RGN mRNA in LUSC tissues. GEPIA website (http://gepia.cancer-pku.cn/) including TCGA and GTEx (https://www.gtexportal.org) databases was used to compare the expression of RGN in LUSC and normal tissues. GSE12428, GSE19188, GSE33532, GSE141479, GSE37745 and GSE29013 datasets in GEO database (https://www.ncbi.nlm.nih.gov/geo/) were applied to obtain RGN expression in normal or tumor tissues. The mRNA profile data in public databases we downloaded was annotated and standardized.

### Differentially expressed single gene

To explore the differential expression of RGN mRNA in different types of tumor and normal tissues, we used the “limma” package in R software to analyze the downloaded data^14^. The “pROC” package was applied to construct a receiver operating characteristic (ROC) prediction model for the normal and LUSC groups. The results were visualized in histogram plots by the R package “ggplot2”.

### Validation of the expression of RGN by immunohistochemistry and qRT-PCR

Ten paraffin-embedded lung squamous cell carcinoma tissues and para-carcinoma tissues from the First Teaching Hospital of Tianjin University of Traditional Chinese Medicine were used for immunohistochemistry (IHC) staining. Experiments involving human tissues were in accordance with the principles of the Declaration of Helsinki and were approved by the Institutional Review Board of the First Teaching Hospital of Tianjin University of Traditional Chinese Medicine. Written informed consent was obtained from all participants. All tissues were incubated overnight with a primary antibody for RGN (1:500, PA5-56057, ThermoFisher) at 4 °C. After washing in Phosphate-Buffered Saline (PBS) for 10 min, each section was incubated by secondary goat anti rabbit antibody for two hours at room temperature. Then, these tissue sections were stained with diaminobenzidine for 3 min and counterstained with hematoxylin. The IHC staining results were analyzed and scored by two investigators assessed in a blinded fashion.

Total RNA was extracted from tissues with TRIzol reagent (Invitrogen, Carlsbad, CA, USA) according to the manufacturer's protocol. NanoDrop 2000 spectrophotometer (Thermo Fisher Scientific, USA) was applied for concentration measurement. Reverse transcription to cDNA was performed according to the instruction of PrimeScript™ RT reagent kit (Takara, Dalian, China). qRT-PCR was carried out in the Q1 Real-Time System (ABI) with SYBR PrimeScript RT-PCR kit (Takara, Dalian, China). The primer sequences were as follows: RGN forward, 5′-AAGATTGAGTGTGTTTTGCCAGA-3′; RGN reverse: 5′-GTCTACAAAGAGCAGAGAGTTGG-3′; β-actin forward: 5′-CTGGCCGTGACCTGACGGAC-3′; β-actin reverse: 5′-GCCTCGGGGCACCTGAACCT-3′. The relative mRNA expression was calculated by the 2^−ΔΔCt^ method.

### Survival and clinical feature analysis

To identify the clinical relevance of RGN, we performed survival analysis based on TCGA-LUSC data. Besides, GEPIA website was used to explore the relationship between RGN and DFS^15^. The correlation between RGN expression and overall survival (OS) in LUSC from GSE3141, GSE37745 and GSE29013 datasets was also evaluated by R package “survival”. Then, the associations between RGN and several clinical features (including age, sex, and tumor stage) were visualized by boxplots. Finally, univariate and multivariate Cox regression analyses were used to assess the prognostic role of RGN and other clinical features.

### Gene set enrichment analysis

Based on the gene expression matrix from TCGA-LUSC dataset, Gene Set Enrichment Analysis (GSEA) was performed to investigate potential biological functions and pathways related to RGN in LUSC^16^. The gene set permutations were performed 1000 times per analysis, and the significantly enriched results were selected with a false discovery rate (FDR) < 0.05 and *P* < 0.05.

### Identification of differentially expressed genes and functional analysis

According to the level of RGN expression, LUSC patients were divided into two groups which were filtered for the identification of differentially expressed genes (DEGs) in TCGA database with the “limma” package. Filtration conditions were log2 fold change (FC)| > 1 and *P* < 0.05. Then, we constructed heatmaps and correlation plots of the 40 DEGs (20 top upregulated genes and 20 top downregulated genes) using the “ggplot2” package. Furthermore, we used the R package “clusterProfiler” for Kyoto Encyclopedia of Genes and Genomes (KEGG) and Gene Ontology (GO) enrichment analyses.

### Construction of a protein–protein interaction network

The protein–protein interaction (PPI) network involving 20 top upregulated genes and 20 top downregulated genes was constructed using the Search Tool for the Retrieval of Interacting Genes (STRING; https://string-db.org/) database. The criteria for selection is the confidence score higher than 0.4. We used Cytoscape 3.8.0. to present the PPI network in which the disconnected nodes were hided and to analyze the subnetworks by the MCODE plugin^17^.

### Construction of the ceRNA regulatory network

The miRNA–RGN and lncRNA–miRNA interaction pairs were obtained from starBase (https://starbase.sysu.edu.cn/), miRDB (https://mirdb.org/), TargetScan (http://www.targetscan.org/) and DIANA-LncBase (http://www.microrna.gr/LncBase)^18–21^. Pearson’s correlation with the criteria of coefficient < − 0.2 and *P* < 0.01 was performed to explore the expression correlation among miRNAs, mRNAs and lncRNAs. Then, differential expression and survival analyses were applied to further screen eligible mRNAs and lncRNAs. The lncRNA-miRNA-mRNA regulatory network was constructed and visualized using Cytoscape 3.8.0.

### Immune clustering analysis

First, the infiltration levels of different immune cell types were quantified by single sample Gene Set Enrichment Analysis (ssGSEA) with R package “GSVA”^22^. We then divided all LUSC samples from TCGA database into high-, middle- and low-immune cell infiltration clusters. Then, we obtained tumor purity, ESTIMATEscore, immunescore and stromalscore of all LUSC samples in the three subtypes by the R package “ESTIMATE”, which reflected the infiltration levels of immune cells and stromal cells within the tumor microenvironment^23^. We also explored the expression of RGN and PD-L1 to validate the difference among three clusters. The R package “ggpubr” was applied to present vioplots. Finally, we used the R package “CIBERSORT” to count the proportion of immune cells of all LUSC samples on the foundation of the three clusters^24^.

### The Tumor IMmune Estimation Resource database analysis

As a comprehensive database for the analysis and visualization of relationship between immune infiltrate levels and several variables, the Tumor IMmune Estimation Resource (TIMER) database (https://cistrome.shinyapps.io/timer/) was applied to determine the relationship between RGN expression levels and immune infiltration based on the CIBERSORT algorithm^25^. We focused on tumor-infiltrating immune cells, including B cells, CD4+ T cells, CD8+ T cells, neutrophils, macrophages, and dendritic cells^26^. SCNA module in the TIMER database was employed to explore the correlation between somatic CNA and abundance of immune infiltration. In addition, we interrogated correlations between RGN expression and gene markers of tumor-infiltrating immune cells in LUSC.

### Prediction of drug response

To predict the half-maximal inhibitory concentration (IC50) of common chemotherapeutic in the high- and low-RGN expression groups in LUSC patients, we used the R package “pRRophetic”^27^. The differences in the IC50 between the two groups were compared by the Wilcoxon signed-rank test, and the results were visualized by boxplots using the R package “ggplot2”. The Cancer Immunome Atlas (TCIA; https://tcia.at/) database was utilized to assess the immune responses of immunotherapy in different RGN expression patients. Finally, the Tumor Immune Dysfunction and Exclusion (TIDE; http://tide.dfci.harvard.edu/) was also applied to evaluate the potential clinical effects of immunotherapy in LUSC.

### Ethics statement

Experiments involving human tissues were in accordance with the principles of the Declaration of Helsinki and were approved by the Institutional Review Board of the First Teaching Hospital of Tianjin University of Traditional Chinese Medicine. Written informed consent was obtained from all participants.

## Results

### Identification of RGN mRNA expression in LUSC and verification by IHC

**Figure 1:**
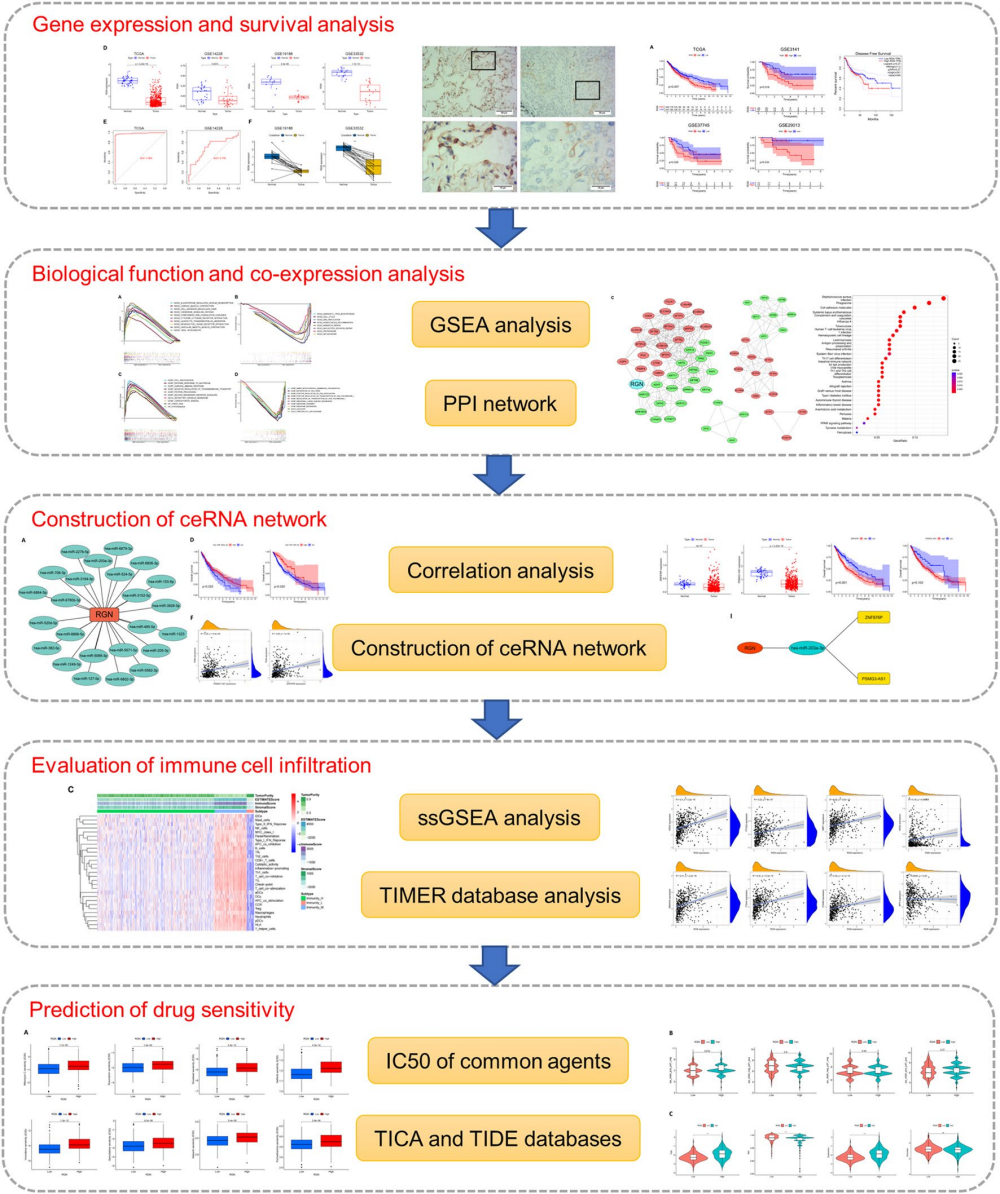
Flow chart for the study design.

**Figure 2:**
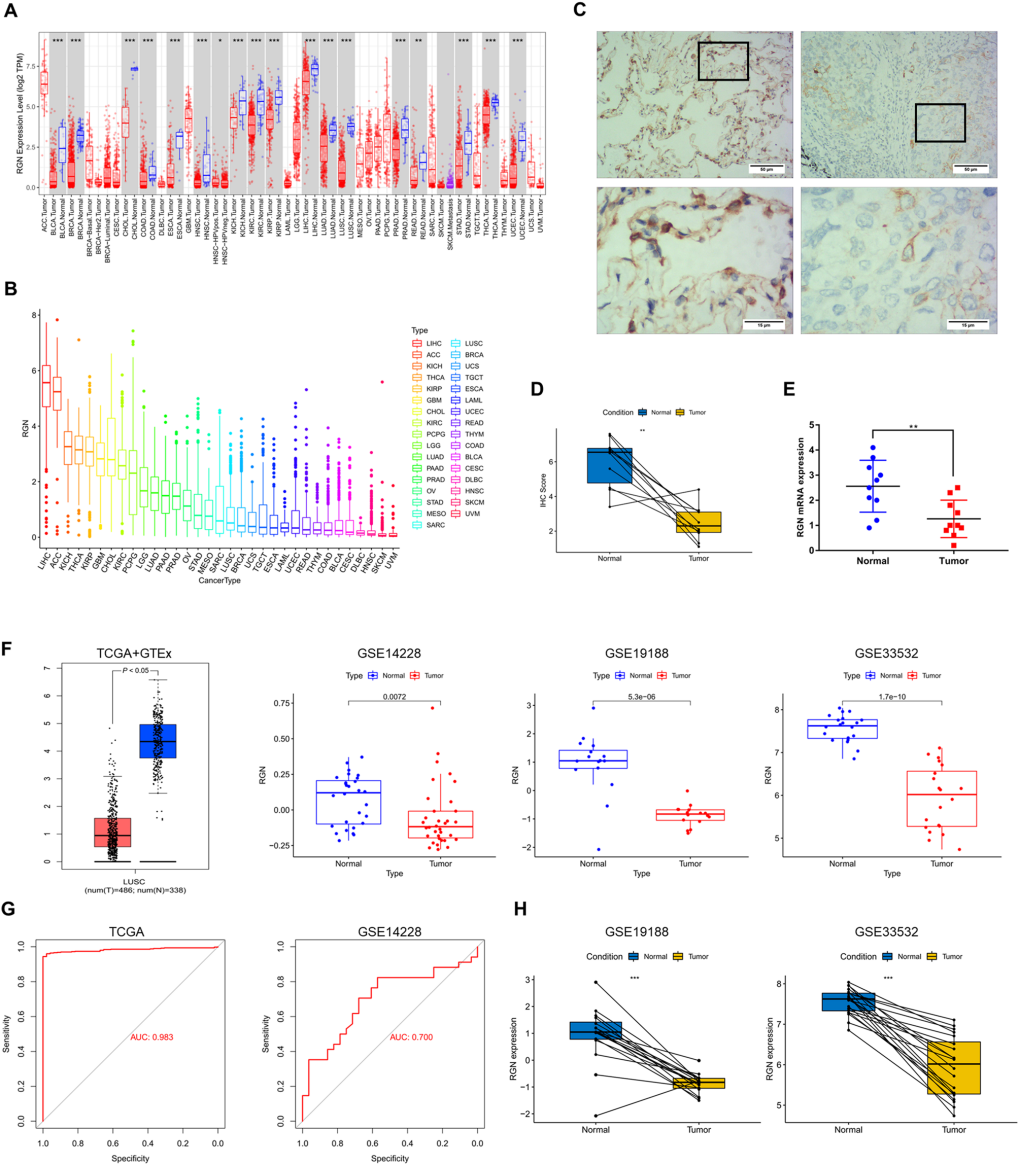
Identification of RGN expression in LUSC from TCGA, GEO databases and clinical specimens. (**A**) RGN expression between normal and tumor tissues in pan-cancer from TCGA database. (**B**) Expression levels of RGN mRNA in pan-cancer. (**C**,**D**) Representative immunohistochemistry images of RGN in LUSC and adjacent non-tumor tissues. The expression level of RGN in tumor tissues was significantly lower than that in adjacent non-tumor tissues. (**E**) RGN mRNA expression in tumor adjacent non-tumor tissues. (**F**) The level of RGN expression was lower in LUSC compared with normal tissues in different datasets. (**G**) Diagnostic value of RGN expression in LUSC in GSE14228 dataset and TCGA database. (**H**) Paired analysis of tumor and normal tissues based on RGN expression in LUSC in GSE19188 and GSE33532 datasets. *P < 0.05; **P < 0.01; ***P < 0.001.

The flowchart of the present research is shown in Fig. 1. In order to explore RGN expression level in various types of tumors, we analyzed the data downloaded from TCGA and GEO databases. In tumor and normal tissues of pan-cancer, mRNA expression of RGN was decreased significantly in seventeen types of cancer, including BLCA, BRCA, HNSC, LIHC, LUAD, LUSC, etc. (Fig. 2A). Based on comparisons of RGN expression in pan-cancers, we summarized that LIHC, ACC, KICH, THCA and KIRP were the top 5 types of cancers with the highest expression (Fig. 2B). Furthermore, LUSC was founded to be associated with a significantly lower expression level of RGN in comparison to normal tissues in RNA-sequencing profile data from public databases (Fig. 2F). We observed that the corresponding area under the ROC curve (AUC) for distinguishing between the LUSC and normal groups was 0.983 in TCGA database and 0.700 in GSE14228 dataset (Fig. 2G). Paired analysis of tumor and normal tissues was used to validate RGN expression in LUSC and normal tissues (Fig. 2H). Finally, we performed IHC and qRT-PCR to evaluate the expression of RGN, and the results presented low RGN protein and mRNA expression level in LUSC tissues compared with adjacent non-tumor tissues (Fig. 2C–E).

### Assessment of prognosis of LUSC patients

**Figure 3:**
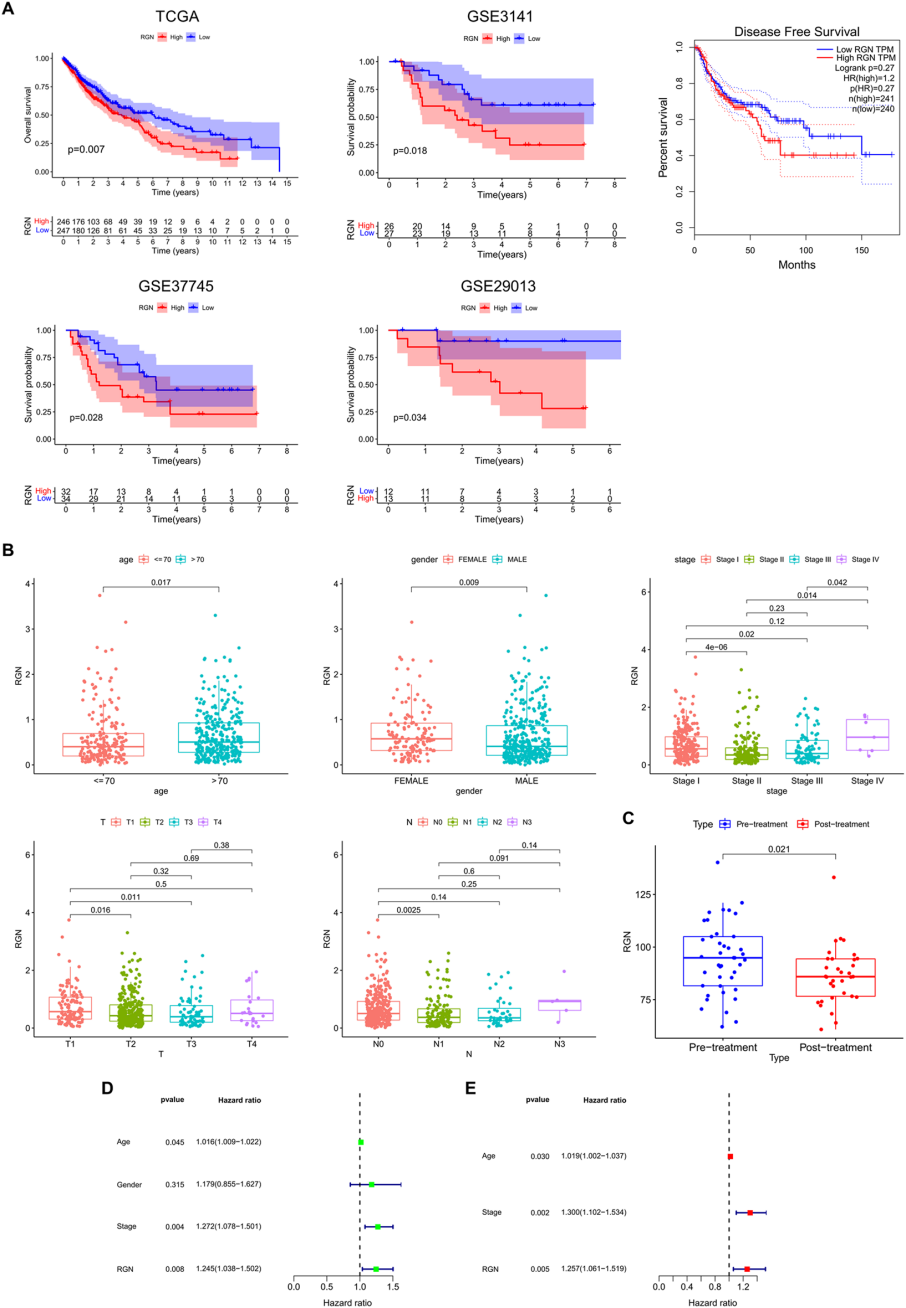
Clinical correlation analysis. (**A**) Kaplan–Meier survival curves of different levels of RGN expression obtained from TCGA, PrognoScan and GEPIA databases. (**B**) RGN expression was significantly correlated with clinical factors, including age, gender, tumor stage, T-stage and N-stage. (**C**) RGN expression was significantly downregulated after immunotherapy. (**D**) Univariate and multivariate Cox regression analyses showed that stage, age and RGN were independent predictors for poor survival in the TCGA-LUSC cohort.

To study the correlation between RGN expression and prognosis of LUSC patients, we analyzed survival data from TCGA and GEO databases and performed survival analysis in GEPIA database. Notably, Kaplan–Meier survival curves exhibited that overexpression of RGN was associated with worse prognosis of LUSC patients in terms of OS. We did not discover significant result related to DFS (Fig. 3A). We performed chi-square test to explore the detailed correlation of RGN expression with a panel of clinical features. As shown in Table 1, RGN expression was closely correlated with age (P = 0.017), gender (P = 0.009), tumor stage (P = 0.001) and pathologic N stage (P = 0.027). The boxplots indicated that female LUSC patients or patients with age greater than 70 years tended to express more significant mRNA expression of RGN. Besides, higher RGN expression was displayed in stage IV, T1 stage and N3 stage (Fig. 3B). Finally, we found that RGN expression was significantly downregulated after immunotherapy (nivolumab) based on GSE141479 dataset (Fig. 3C, P = 0.021). Univariate and multivariate Cox regression analyses showed that stage, age and RGN were independent predictors for poor survival in the TCGA-LUSC cohort (Fig. 3D,E).

**Table 1:** Correlation between RGN mRNA expression and clinicopathologic features in TCGA database.

Clinical features	RGN expression		*P*-value
	Low (%)	High (%)	
Age			
≤ 70	116 (42.8)	90 (33.2)	0.017*
> 70	155 (57.2)	181 (66.8)	
Gender			
Female	57 (21.0)	85 (31.4)	0.009*
Male	214 (79.0)	186 (68.6)	
Race			
White	245 (90.4)	244 (90.0)	0.633
Black/African American	22 (8.1)	20 (7.4)	
Asian	4 (1.5)	7 (2.6)	
Laterality			
Bilateral	247 (89.8)	241 (87.3)	0.424
Left	12 (4.4)	11 (4.0)	
Right	16 (5.8)	24 (8.7)	
Tumor stage			
Stage I	111 (41.0)	160 (59.0)	0.001*
Stage II	109 (40.2)	66 (24.4)	
Stage III	49 (18.1)	39 (14.4)	
Stage IV	2 (0.7)	6 (2.2)	
Pathologic M stage			
M0	267 (98.5)	262 (96.7)	0.160
M1	4 (1.5)	9 (3.3)	
Pathologic N stage			
N0	160 (59.0)	189 (69.7)	0.027*
N1	84 (31.0)	55 (20.3)	
N2	26 (9.6)	17 (6.3)	
N3	1 (0.4)	10 (3.7)	
Pathologic T stage			
T1	53 (19.6)	70 (25.8)	0.085
T2	164 (60.5)	156 (57.6)	
T3	42 (15.5)	33 (12.2)	
T4	12 (4.4)	12 (4.4)	
Radiation therapy			
Yes	35 (12.9)	28 (10.3)	0.348
No	236 (87.1)	243 (89.7)	
New tumor event after initial treatment			
Yes	71 (26.2)	74 (27.3)	0.771
No	200 (73.8)	197 (72.4)	
Cigarettes per day exposures			
≤ 3	156 (57.6)	177 (65.3)	0.064
> 3	115 (42.4)	94 (34.7)	
Prior malignancy diagnosis			
Yes	32 (11.8)	34 (12.5)	0.793
No	239 (88.2)	237 (87.5)	

*P < 0.05.

### Functional enrichment analysis

**Figure 4:**
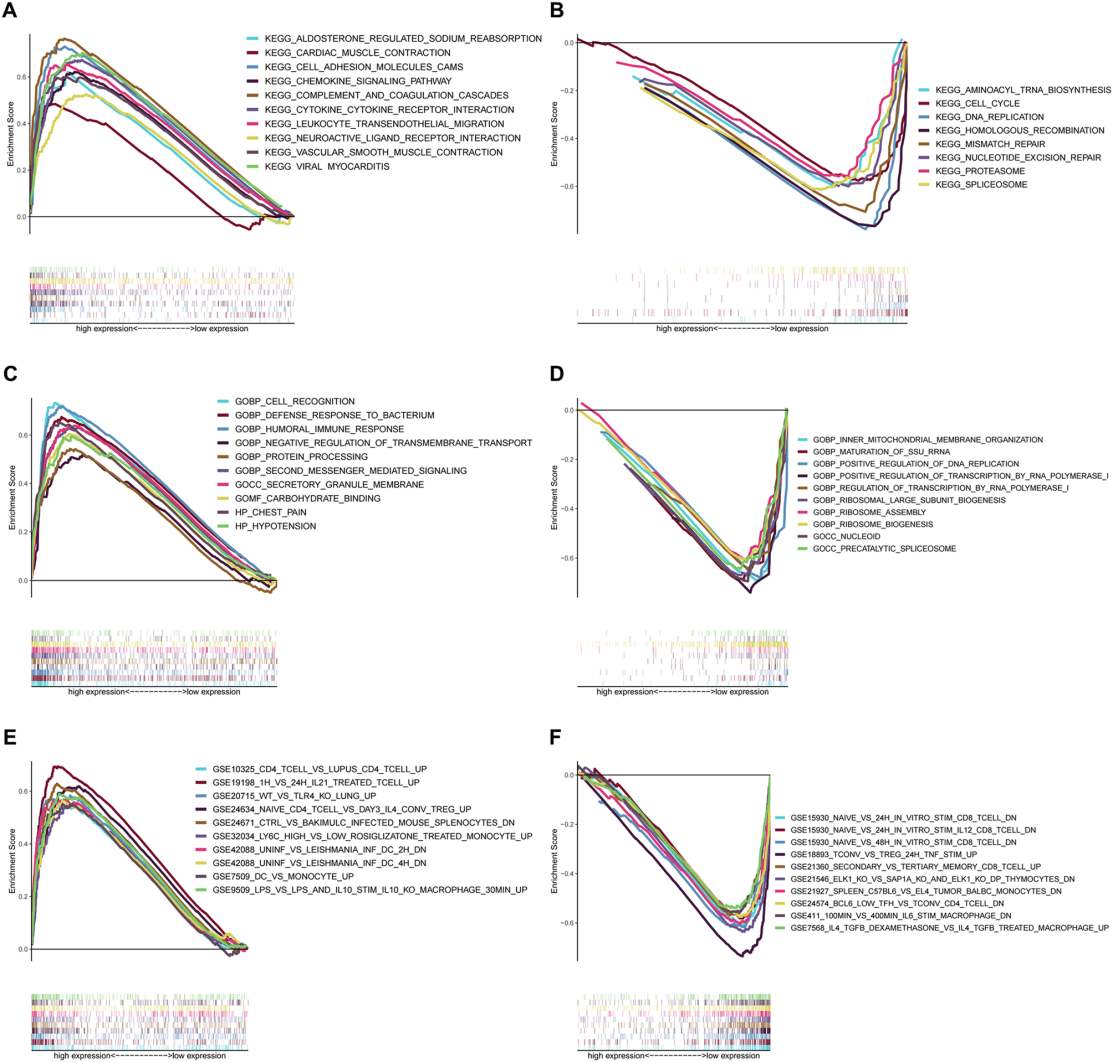
GSEA for LUSC samples with high and low RGN expression. (**A**,**B**) The enriched gene sets in KEGG by samples with high and low RGN expression. (**C**,**D**) The enriched gene sets in GO with samples with high and low RGN expression. (**E**,**F**) The enriched gene sets related to immunologic signature with samples with high and low RGN expression.

To examine the functional enrichment of different expression levels of RGN, GSEA was performed between the RGN low group and RGN high group in LUSC patients from the TCGA cohort. Immunologic signature, GO and KEGG gene sets were taken as references. KEGG and GO enrichment terms exhibited that RGN expression was positively correlated with chemokine signaling pathway, cytokine–cytokine receptor interaction, cell recognition, humoral immune response, etc. The global expression changes produced in LUSC patients were negatively correlated with cell cycle, DNA replication, ribosome assembly, nucleoid, etc. (Fig. 4A–D). Additionally, GSEA also revealed an association with immunologic signature terms, including “GSE10325 CD4 T-cell vs lupus CD4 T-cell up”, “GSE19198 1 h vs 24 h IL21 treated T-cell up”, “GSE15930 naïve vs 24 h in vitro stim CD8 T-cell down”, “GSE18893 tconv vs treg 24 h TNF stim up”, etc. (Fig. 4E,F).

### Analysis of DEGs grouped according to RGN expression

**Figure 5:**
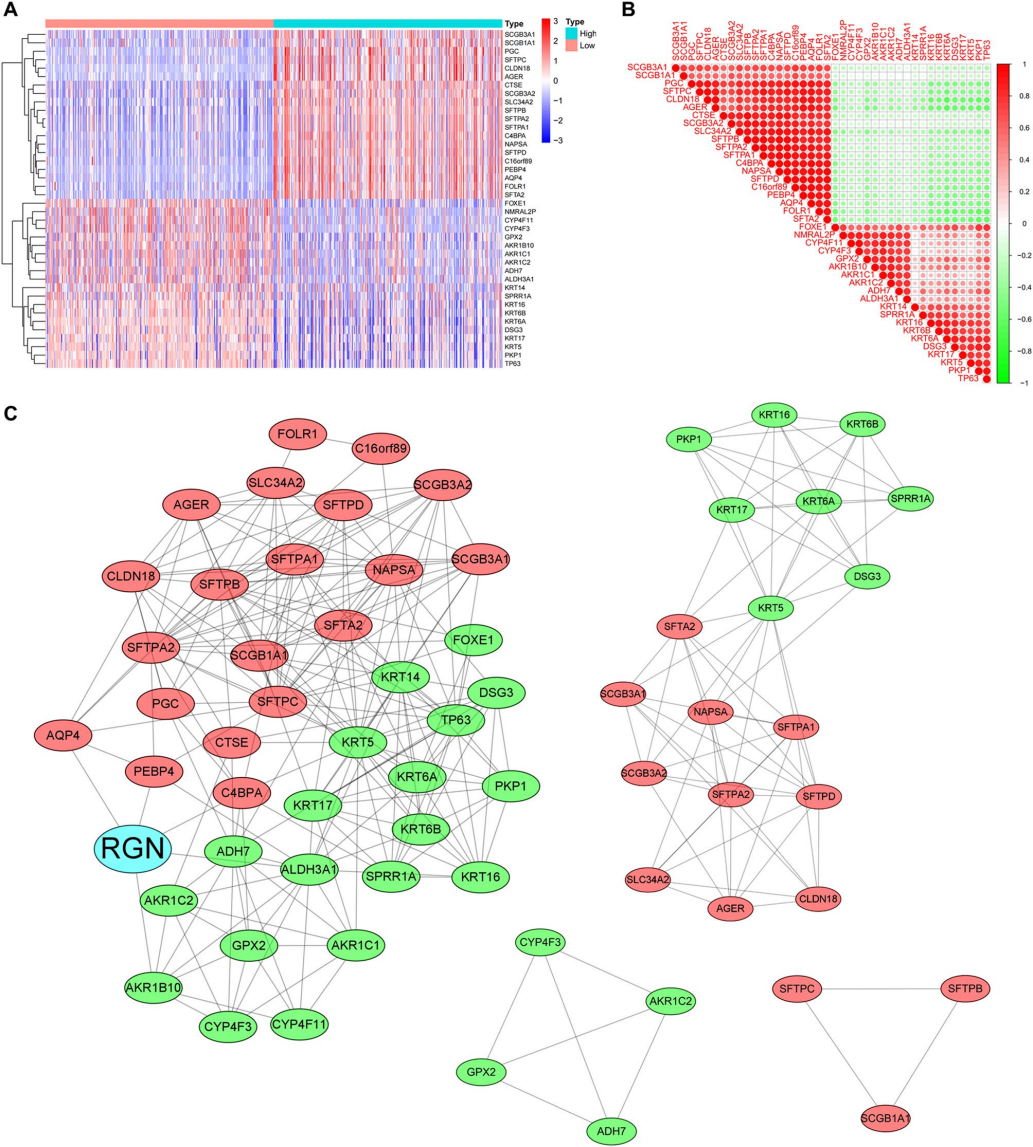
Analysis of DEGs between high and low RGN expression groups. (**A**) Heatmap of the top 20 upregulated and top 20 downregulated DEGs between the high and low RGN expression groups of LUSC samples in TCGA database; red dots indicate high expression, whereas blue dots indicate low expression. (**B**) Correlations of expression level among the top 20 upregulated and top 20 downregulated DEGs. (**C**) The PPI network of RGN and DEGs.

**Figure 6:**
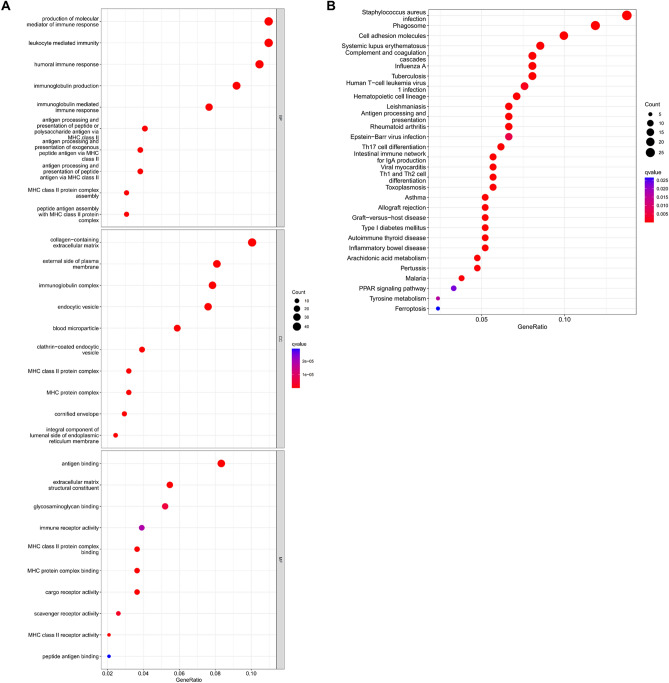
GO and KEGG pathway enrichment analysis. (**A**) The bar plot of KEGG pathway enrichment analysis (BP, biological process; CC, cell component; MF, molecular function). (**B**) The bar plot of GO pathway enrichment analysis.

According to the high and low RGN expression groups, we identified a total of 87 downregulated genes and 335 upregulated genes (|log2FC| > 1 and *P* < 0.05) in TCGA database (Supplementary Table 1). Then, we selected separately the top 20 genes in downregulated and upregulated groups to exhibit gene correlations and expression levels (Fig. 5A,B). In addition, we constructed a PPI network and its subnetworks based on the STRING database using the Cytoscape software (Fig. 5C). Finally, GO and KEGG functional enrichment analysis were performed to distinct the functions of DEGs. The results demonstrated that the DEGs were associated with leukocyte mediated immunity, immunoglobulin production, endocytic vesicle, antigen binding, staphylococcus aureus infection, phagosome, etc. (Fig. 6).

### Construction of prognosis-related ceRNA network

**Figure 7:**
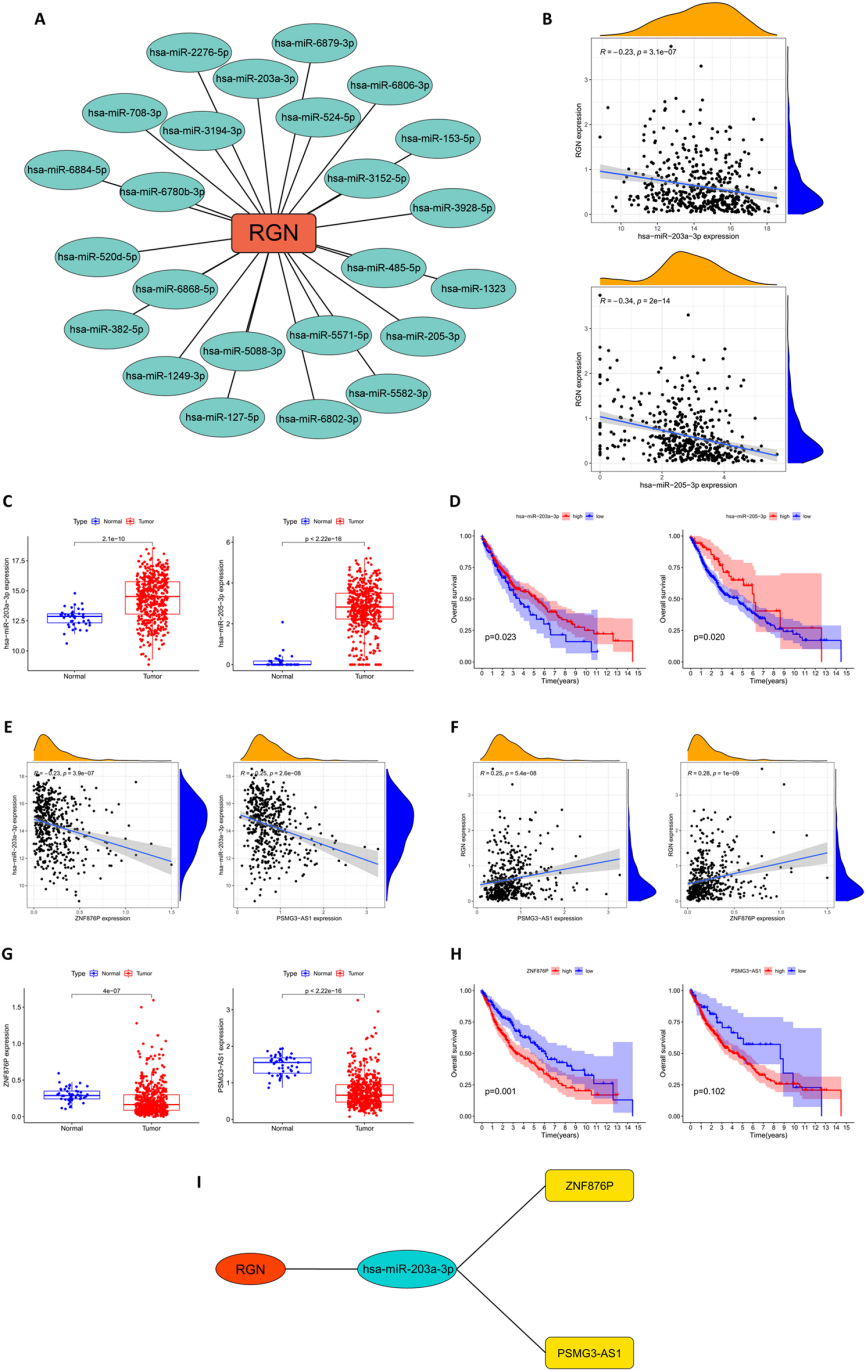
Construction of prognosis-related ceRNA network. (**A**) RGN was closely associated with 24 miRNAs. (**B**) The correlation analysis of RGN and two target miRNAs (miR-203a-3p and miR-205-3p). (**C**) The expressions of miR-203a-3p and miR-205-3p in LUSC and normal tissues. (**D**) Kaplan–Meier curve analysis of miR-203a-3p and miR-205-3p for the overall survival in LUSC patients. (**E**) Two target lncRNAs (ZNF876P and PSMG3-AS1) were negatively correlated with miR-203a-3p. (**F**) ZNF876P and PSMG3-AS1 were positively correlated with RGN. (**G**) The expression of ZNF876P and PSMG3-AS1 in LUSC and normal prostate tissues. (**H**) Kaplan–Meier curve analysis of ZNF876P and PSMG3-AS1 for the overall survival in LUSC patients. (**I**) The diagram of the final ceRNA network.

Based on the results of starBase, miRDB and TargetScan databases, we screened 24 miRNAs which were associated with the expression of RGN (Fig. 7A). Then, correlation, survival and differential expression analyses were performed to further select eligible miRNAs for subsequent analyses. We found that miR-203a-3p and miR-205-3p were negatively correlated with RGN expression (Fig. 7B). Importantly, the expression of these miRNAs in LUSC tissues was significantly higher than that in normal tissues (*P* < 0.05) (Fig. 7C). Besides, Kaplan–Meier curves presented that higher expression of miR-203a-3p or miR-205-3p was significantly associated with longer OS of LUSC patients (Fig. 7D).

The lncRNAs related to miR-203a-3p or miR-205-3p were obtained from starBase and DIANA-LncBase databases. Correlation analyses revealed that ZNF876P and PSMG3-AS1 were negatively correlated with miR-203a-3p, while no genes significantly related to miR-205-3p were observed (Fig. 7E). Additionally, miR-203a-3p and miR-205-3p were negatively correlated with RGN (Fig. 7F). As shown in Fig. 7G, the expression of ZNF876P and PSMG3-AS1 were downregulated in LUSC tissues compared with normal tissues. Furthermore, survival analyses showed that lower expression of ZNF876P was significantly associated with longer OS of LUSC patients. Although the survival curve of PSMG3-AS1 did not show significant difference, it can be observed that the low expression group before 9 years also had a longer survival period (Fig. 7H). Therefore, based on the ceRNA hypothesis, we constructed the ceRNA network about RGN (Fig. 7I).

### Construction and validation of LUSC clustering

**Figure 8:**
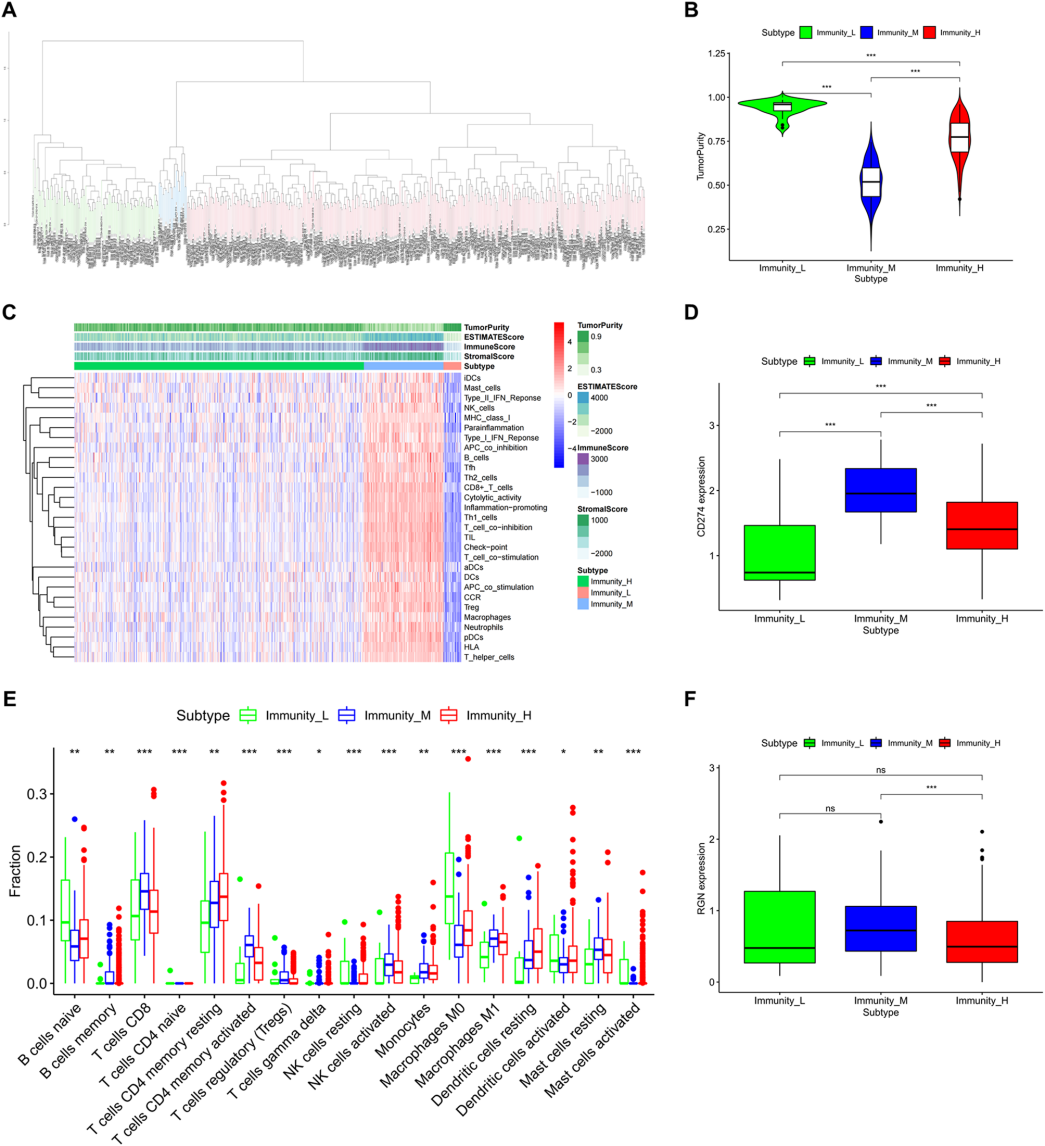
Construction and validation of LUSC clustering. (**A**) All LUSC samples in TCGA database were divided into three clusters based on immune infiltration level. (**B**) The violin plot of the difference in Tumor Purity among high-, middle-, and low-immune cell infiltration cluster. (**C**) The enrichment levels of 29 immune-related cells and types in the high-, middle-, and low-immune cell infiltration cluster. The Tumor Purity, ESTIMATE Score, Immune Score and Stromal Score of every patient gene were showed combine with the clustering information. (**D**) The relationship between PD-L1 expression and the three subtypes. (**E**) The proportion of immune cells among the high-, middle-, and low-immune cell infiltration cluster using CIBERSORT algorithm. (**F**) The relationship between RGN expression and the three subtypes. *P < 0.05; **P < 0.01; ***P < 0.001; *ns* not significant.

We used ssGSEA method for the RNA-sequencing data of LUSC samples downloaded from TCGA database to examine the immune cells infiltration, and we obtained the abundance levels of 29 immune-related cells and types in LUSC samples. Then, all LUSC samples were assigned into high-, middle-, and low-immune cell infiltration clusters based on immune infiltration evaluated by unsupervised hierarchical clustering algorithm (Fig. 8A). Importantly, we calculated Stromal Score, Immune Score, ESTIMATE Score and Tumor Purity by ESTIMATE algorithm for validating the applicability of the clustering result. Our results revealed that Stromal Score, Immune Score, and ESTIMATE Score in low immune cell infiltration cluster were lower than that of other two clusters, but Tumor Purity at a relatively high level in low group (Fig. 8C). The violin plot also shown that the degree of Tumor Purity was highest in low immune cell infiltration cluster (Fig. 8B). Similarly, the boxplot further presented immune infiltration of immune-related cells and types in the three clusters by CIBERSORT algorithm (Fig. 8E). Furthermore, we discovered that PD-L1 expression was significantly different in low-, middle-, high- immune cell infiltration clusters, and RGN expression showed significant difference between middle and high groups (Fig. 8D,F).

### Immune cell infiltration analysis

**Figure 9:**
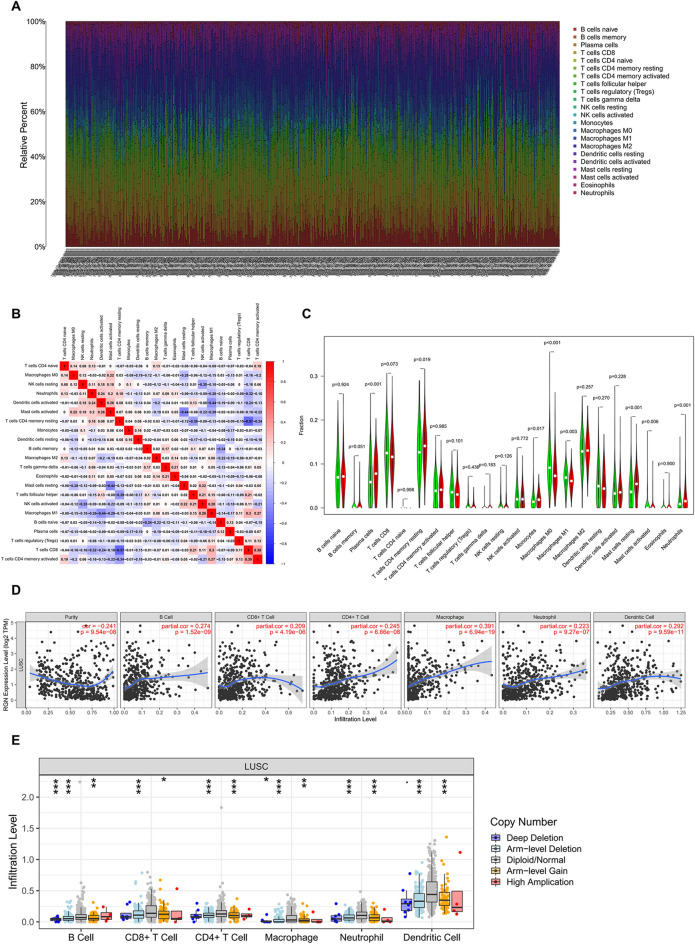
Correlation analysis between RGN expression and immune cells in LUSC. (**A**) The ratio of 22 types of tumor immune infiltrating cells in LUSC samples. (**B**) The correlation among 22 immune cell types in the LUSC group. (**C**) Comparison of the level of the 22 infiltrating immune cell types and RGN between the high- and low-expression groups; red reflects high expression, whereas green reflects low expression. (**D**) Scatterplots of correlations between RGN expression and immune cells, including CD4+ T cell, macrophage, B cell, CD8+ T cell, neutrophil or dendritic cell. (**E**) The correlation between somatic copy number variation and immune infiltration levels of six immune cells in LUSC.

**Figure 10:**
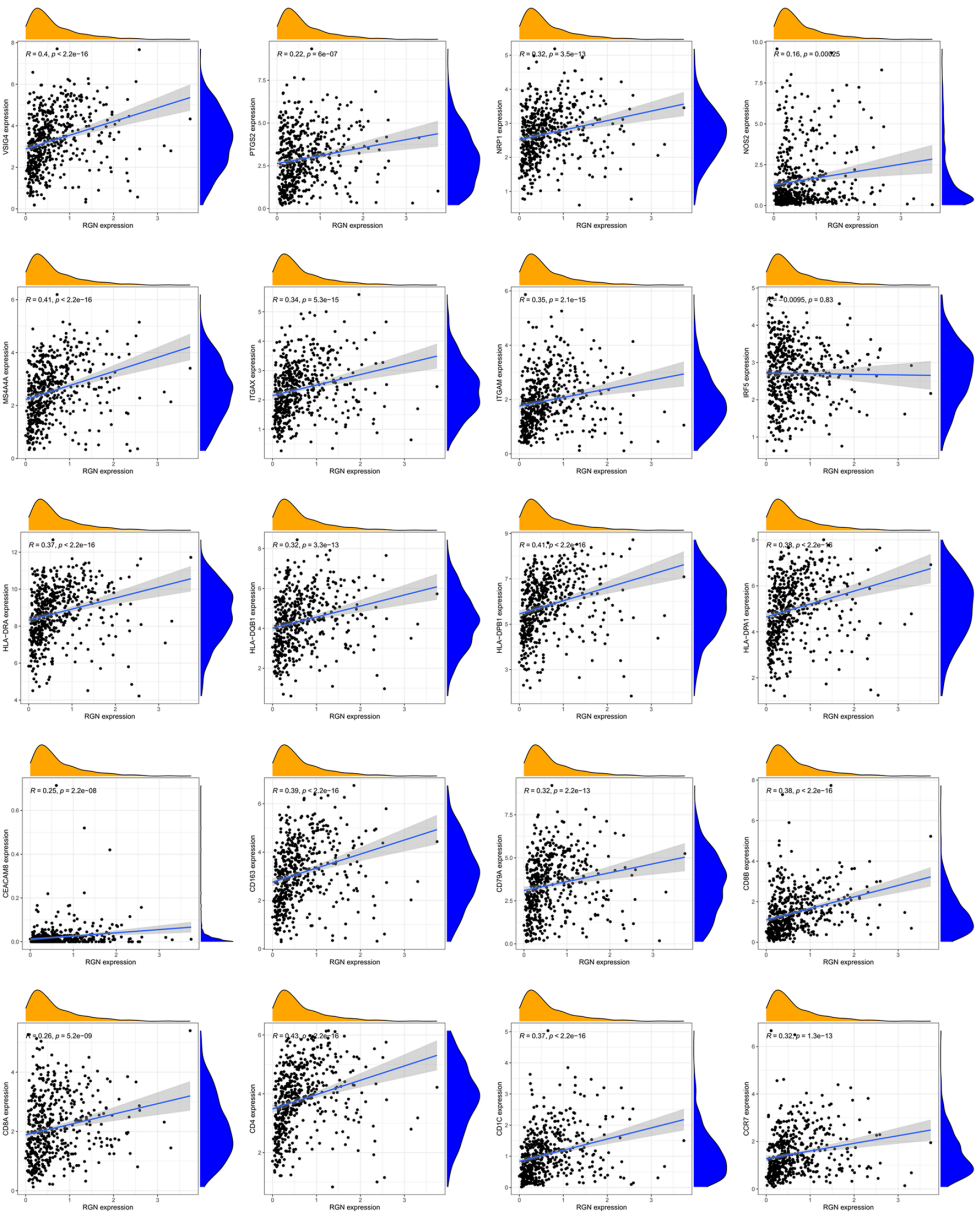
The mRNA expression of RGN was related to gene markers of immune cells.

In order to identify the correlation between the immune microenvironment and RGN expression, we built a heatmap containing the ratio of 22 types of tumor immune infiltrating cells (Fig. 9A), and confirmed the correlation among 22 immune cell types in the LUSC group (Fig. 9B). The vioplot showed expression levels of different immune cells in RGN low profile and RGN high profile in LUSC patients, and we observed significant differences in Plasma cells, T cells CD4 memory resting, Macrophages M0, Macrophages M1, Mast cells resting, Mast cells activated and Neutrophils (Fig. 9C). In addition, we applied the TIMER database to estimate the correlations of RGN expression with immune cell infiltration. It was obviously that RGN expression was positively correlated with immune infiltration of B cell, CD8+ T cell, CD4+ T cell, Macrophage, Neutrophil and Dendritic cell (Fig. 9D). We also explored the comparison of tumor infiltration levels in LUSC with different somatic copy number alterations for RGN. The results showed that the infiltration of Dendritic cell was associated with the high level of copy number alterations for RGN in LUSC (Fig. 9E). Finally, we investigated the correlations of RGN expression with the markers of six immune cells in LUSC, including B cell markers (CD19, CD79A), CD8+ T cells markers (CD8A, CD8B), CD4+ T cell markers (CD4), M1 macrophage markers (NOS2, IRF5, PTGS2), M2 macrophage markers (VSIG4, MS4A4A), neutrophil markers (CEACAM8, ITGAM, CCR7) and dendritic cell markers (HLA-DPB1, HLA-DQB1, HLA-DRA, HLA-DPA1, CD1C, NRP1, ITGAX). Our results revealed that significant positive correlations between RGN expression and these markers except IRF5 (Fig. 10, Supplementary Table 2).

### Prediction of drug response to chemotherapy and immunotherapy

**Figure 11:**
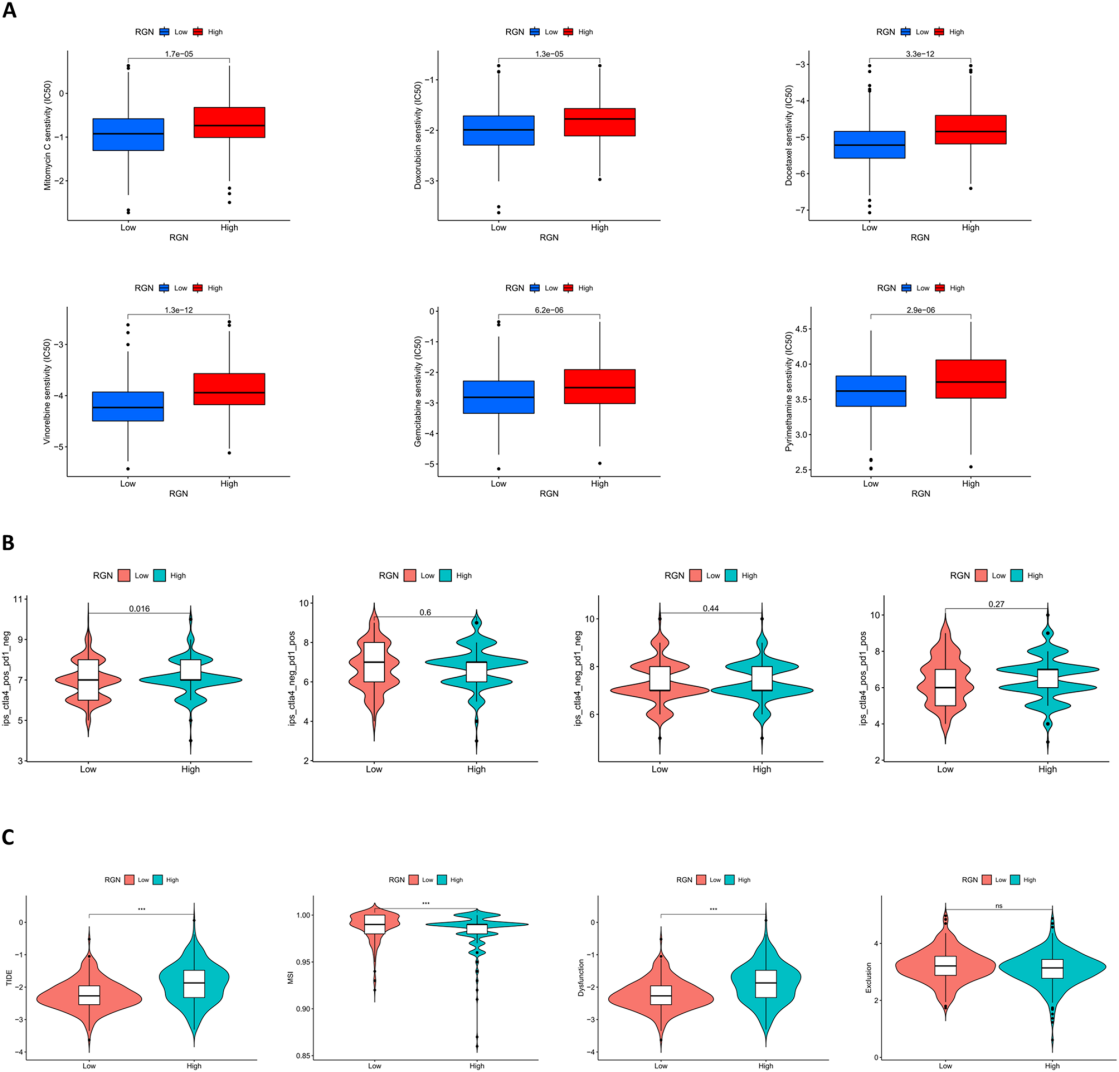
Prediction of RGN-related drug responses in LUSC patients. (**A**) Prediction of the IC50 of common therapeutic agents in high- and low-RGN expression patients. (**B**) Distribution of RGN expression in CTLA4 or PD1 scores by TCIA database. (**C**) Distribution of RGN expression in TIDE, MSI, dysfunction and exclusion scores by TIDE database. ***P < 0.001; *ns* not significant.

The pRRophetic algorithm was used to predict the IC50 of common therapeutic in high- and low-RGN expression patients. As shown in Fig. 11A, several chemotherapeutic drugs including docetaxel and gemcitabine had lower IC50 in the low expression group, indicating that these patients were more sensitive to this chemotherapeutic drug. According to the data mining from the TCIA database, the results indicated that low- and high-RGN expression groups had significant difference in response to immune checkpoint inhibitors targeting CTLA-4. However, these groups did not show differences in the prediction of PD1-targeted immunotherapy (Fig. 11B). Furthermore, we used the TIDE database to assess the potential clinical effects of immunotherapy in different RGN expression groups. As a result, high expression group presented a higher TIDE score, a higher dysfunction score, and a lower MSI score, which demonstrated that these patients tended to have a lower efficacy after accepting the immunotherapy than LUSC patients with low RGN expression (Fig. 11C).

## Discussion

As an important regulatory protein in Ca^2+^-dependent signaling pathway, RGN expression was regulated by extracellular and intracellular calcium levels. Protein kinase C (PKC) pathway was able to enhance nuclear localization of RGN for modestly promoting gene expression^28,29^. For liver cancer, researchers have noticed that RGN expression was also induced by various hormonal stimulation, including calcitonin, insulin and estrogen. Additionally, it was reported that the consequences of the aging process decreased liver RGN expression^30^.

In the course of tumor development, RGN could exert complex biological cell functions and show antitumor activity. RGN expression was associated with preventive effects against the proliferation of tumor and progression of cancer cells^31^. Similarly, RGN was also found to play a role as a suppressor protein in carcinogenesis, and high expression of endogenous RGN was suggested to reveal preventive and therapeutic effects on carcinogenesis^32^. Overexpression of RGN can suppress bone metastatic activity of MDA-MB-231 human breast cancer cells^33^. A recent study has demonstrated that RGN could promote dormancy of prostate cancer, and patients expressing higher level of RGN could show significantly longer recurrence-free survival (RFS) and OS^34^. The effects of RGN include activation of p38 MAPK and inhibition of Erk signaling pathway. Besides, secretory miR-23c level in the exosomes was upregulated by RGN to suppress angiogenesis.

In our study, we explored the biological landscape of RGN in LUSC in novel dimensions. For the first time, we discovered that RGN expression was significantly downregulated in tumor tissues compared with normal tissues and closely related to clinical factors and prognosis in LUSC patients according to TCGA and GEO databases. On the foundation of the expression of RGN mRNA, we identified DEGs which were mostly involved in immunobiological processes such as humoral immune response and leukocyte mediated immunity. Then, we found that RGN was related to tumor-infiltrating immune cells in LUSC. Meanwhile, the TIMER database provided an estimation of the immune infiltration levels. To our knowledge, our analyses provide novel insights into the prognostic role of RGN and potential role of RGN in the tumor immunology of LUSC.

Previous studies pointed out that RGN expression played a role in regulating various oncogenes and tumor suppressors by exerting growth suppressive effect^35^. It was noting that high RGN expression was closely associated with prolonged survival of patients with cancers including prostate cancer, lung adenocarcinoma and colorectal cancer^36–38^. We founded that RGN was differentially expressed in normal and tumor tissues and associated with prognosis of LUSC patients. Intriguingly, RGN expression was at a lower level in tumor samples compared to normal samples, but our Kaplan–Meier survival curves demonstrated that LUSC patients with high RGN expression tended to have poor survival. Our controversial findings may confuse the role of RGN in the initiation and subsequent progression of LUSC. In terms of mechanism, RGN is in a pathway that may be enhanced or otherwise regulated in LUSC. A study has revealed that HIF-1α protein binds directly to the HRE binding motifs within the RGN promoter to regulate the the expression of RGN^39^. The expression of HIF-1α was significantly higher in LUSC than those in normal lung tissues and related to worse survival time^40,41^. Therefore, we speculated the upregulated RGN with a worse prognosis in our research might be the result of regulation of high levels of HIF-α, especially in the hypoxic tumor microenvironment after tumor formation. Besides, instead of functioning in isolation, RGN presents different expression levels at various stages of disease and produces biological effects by complex regulatory mechanisms including non-coding RNAs and epigenetic modifications. A study presented that epigenetic re-programming of RGN in NSCLC led to reduced expression of Regucalcin^42^. In summary, owing to lack of sufficient related studies, it is worth performing relevant experiments to explore specific mechanisms for further explaining the role of RGN in LUSC.

Recently, considering the importance of immune microenvironment in the progression of cancer and the efficacy of individualized immunotherapy, many biological and clinical researches have focused on TIME^43,44^. As a vital factor in tumor development, the immune landscape can be explored to identify more genomic and clinical characteristics in different immune infiltration patterns. In our study, we divided the LUSC samples into three clusters based on the enrichment of 29 immune cell types. Then, we found that Stromal Score, Immune Score, and ESTIMATE Score calculated by ESTIMATE algorithm in low immune cell infiltration cluster were lower than that of other two clusters, but Tumor Purity at a relatively high level in low group. Besides, there were also obviously difference in the proportion of 17 immune cells in three clusters using CIBERSORT algorithm. We also discovered that PD-L1 and RGN expression was significantly different in low-, middle-, high- immune cell infiltration clusters, which could validate the heterogeneity of immune microenvironment in LUSC. By mining the TIMER database, we further revealed that RGN expression was positively correlated with high levels of immune cell infiltration such as macrophages. Tumor islet-infiltrating M2 macrophages influence the prognosis of NSCLC patients, and the analysis of M2 macrophages and PD-L1 in combination may enhance the accuracy of prognostic prediction^45^. In addition, positive correlations were observed between RGN expression and certain immunological markers including CD4, CD8, CD163, CCR7, CD1C, etc. The tumor microenvironment of tumorigenesis was particular rich in CD163+ macrophages^46^. Researchers discovered that preoperative prognostic nutritional index level was associated with CD4+ T and CD8+ T lymphocyte infiltration status in patients with surgically resected LUSC^47^. Furthermore, GO and KEGG analysis showed that the DEGs grouped by RGN expression level were associated with leukocyte mediated immunity, immunoglobulin production and humoral immune response, which also displayed strong correlations between RGN and immune infiltration in LUSC.

The research evidence of RGN in immune microenvironment is relatively insufficient. A study has demonstrated that RGN could exert anti-inflammatory effects on adipocyte cocultured with macrophages^48^. Our results also observed that the high RGN expression group often showed the low level of macrophage infiltrations, including macrophage M0 and M1. Under the stimulation of IFN-γ or other factors, macrophage M0 was polarized into macrophage M1 and produced a large amount of pro-inflammatory factors, including IL-1, IL-6 and TNF-α, etc. These inflammatory cells and related inflammatory mediators in TIME may contribute to tumorigenesis and cancer progression^49^. Therefore, RGN may regulate TIME by influencing and/or interacting with macrophages in LUSC, which was a possible mechanism. Our analysis implied that RGN may be important for regulating immune cell infiltration and activation in tumor microenvironment of LUSC, and further study was urged to investigate role of various immune cells and types in tumor microenvironment.

Notably, there are several inevitable limitations existing in our study. The data we used were mainly obtained from TCGA and GEO database, therefore we could not validate the prognostic role of RGN from other databases or own tissue samples. Additionally, further in vivo and in vitro studies are urged to investigate the potential biological function of RGN and the detailed mechanism by which these significant immune cells participated in LUSC progression.

## Conclusion

Our study provides novel insights into the potential role of RGN in LUSC. RGN is downregulated in LUSC and considered to be closely associated with LUSC prognosis. We also constructed a prognostic ceRNA network related to RGN. Importantly, RGN expression is strongly correlated with immune infiltration in LUSC. Although further systematic experimental studies are required, our findings showed that RGN could play an important role in the infiltration of immune cells and be a promising prognosing biomarker to improve the current therapeutic practice of LUSC.

## Supplementary Information

Supplementary Table 1.

Supplementary Table 2.

## Data Availability

The datasets presented in this study can be found in online repositories. The names of the repository/repositories and accession number(s) can be found below: The Cancer Genome Atlas (TCGA; https://portal.gdc.cancer.gov/); GEPIA (http://gepia.cancer-pku.cn/); GTEx (https://www.gtexportal.org); Tumor Immune Estimation Resource (TIMER; (https://cistrome.shinyapps.io/timer/); The STRING (https://string-db.org/); The starBase (https://starbase.sysu.edu.cn/); miRDB (https://mirdb.org/); TargetScan (http://www.targetscan.org/); DIANA-LncBase (http://www.microrna.gr/LncBase); The Cancer Immunome Atlas (TCIA; https://tcia.at/) and the Tumor Immune Dysfunction and Exclusion (TIDE; http://tide.dfci.harvard.edu/).
