# Supplementary material for: RGN as a prognostic biomarker with immune infiltration and ceRNA in lung squamous cell carcinoma
Source: Sci Rep. 2023 May 9;13:7553. doi: 10.1038/s41598-023-32217-z (PMC10170118; doi:10.1038/s41598-023-32217-z)
Supplement: Supplementary file 1 — Supplementary Table 1. [file 41598_2023_32217_MOESM1_ESM.docx]

Supplementary table 1：Identification of differentially expressed genes

| id | logFC | AveExpr | t | P.Value | adj.P.Val | B |
| --- | --- | --- | --- | --- | --- | --- |
| RGN | 1.083913 | 0.80171 | 23.82648 | 1.11E-86 | 4.28E-82 | 186.8535 |
| C7 | 2.13213 | 2.685786 | 16.69841 | 6.43E-51 | 1.24E-46 | 105.0228 |
| AOC3 | 1.672968 | 2.794437 | 16.22415 | 1.22E-48 | 1.57E-44 | 99.81242 |
| SELENBP1 | 1.895496 | 3.215885 | 15.95249 | 2.40E-47 | 2.32E-43 | 96.85141 |
| C4BPA | 2.590505 | 2.878099 | 15.85574 | 6.92E-47 | 5.34E-43 | 95.80115 |
| NDNF | 1.885842 | 2.013617 | 15.7673 | 1.81E-46 | 1.08E-42 | 94.84323 |
| A2M | 1.789039 | 6.113525 | 15.7605 | 1.95E-46 | 1.08E-42 | 94.76969 |
| INMT | 1.778056 | 1.881441 | 15.56462 | 1.64E-45 | 7.92E-42 | 92.65554 |
| MGP | 1.660978 | 4.940579 | 15.55304 | 1.86E-45 | 7.98E-42 | 92.5309 |
| AQP4 | 2.189285 | 2.082897 | 15.37513 | 1.27E-44 | 4.54E-41 | 90.62013 |
| PDK4 | 1.65516 | 1.90829 | 15.37367 | 1.30E-44 | 4.54E-41 | 90.60446 |
| SFTPD | 2.997182 | 4.24699 | 15.35546 | 1.58E-44 | 5.07E-41 | 90.40938 |
| MFAP4 | 2.00364 | 4.485679 | 15.27512 | 3.75E-44 | 1.11E-40 | 89.54987 |
| ADGRF5 | 1.56045 | 2.736237 | 15.25313 | 4.75E-44 | 1.31E-40 | 89.31491 |
| VSIG2 | 1.718982 | 2.009445 | 15.12188 | 1.94E-43 | 5.00E-40 | 87.91525 |
| COX4I2 | 1.195024 | 2.315399 | 15.04998 | 4.20E-43 | 9.53E-40 | 87.15069 |
| PEBP4 | 2.132154 | 1.964055 | 15.01383 | 6.18E-43 | 1.32E-39 | 86.76685 |
| AQP1 | 1.831596 | 4.984503 | 14.98986 | 7.98E-43 | 1.62E-39 | 86.51256 |
| CRTAC1 | 1.790881 | 2.036265 | 14.9609 | 1.09E-42 | 2.10E-39 | 86.20555 |
| FOLR1 | 2.235608 | 2.727481 | 14.87635 | 2.68E-42 | 4.70E-39 | 85.31055 |
| CSF3R | 1.217328 | 1.893458 | 14.83211 | 4.29E-42 | 6.89E-39 | 84.84314 |
| C16orf89 | 2.156671 | 2.519809 | 14.71984 | 1.41E-41 | 1.82E-38 | 83.6596 |
| ADH1B | 1.864458 | 1.534343 | 14.68344 | 2.08E-41 | 2.59E-38 | 83.27666 |
| CLEC14A | 1.187016 | 2.938431 | 14.64438 | 3.14E-41 | 3.56E-38 | 82.86621 |
| ESAM | 1.181476 | 3.219849 | 14.63002 | 3.66E-41 | 4.03E-38 | 82.71543 |
| SFTA2 | 2.58178 | 2.907426 | 14.62455 | 3.87E-41 | 4.15E-38 | 82.65803 |
| CCDC69 | 1.134709 | 2.662499 | 14.60593 | 4.71E-41 | 4.92E-38 | 82.46273 |
| LRRK2 | 1.465334 | 1.689631 | 14.586 | 5.82E-41 | 5.91E-38 | 82.25375 |
| PECAM1 | 1.295227 | 4.12074 | 14.57875 | 6.28E-41 | 6.22E-38 | 82.17774 |
| LMO3 | 1.224326 | 1.138542 | 14.50906 | 1.31E-40 | 1.26E-37 | 81.44809 |
| TMEM125 | 1.60285 | 2.806041 | 14.50651 | 1.35E-40 | 1.27E-37 | 81.42146 |
| NAPSA | 2.908953 | 4.096623 | 14.44743 | 2.51E-40 | 2.30E-37 | 80.80413 |
| SLC34A2 | 2.765151 | 4.695696 | 14.42239 | 3.26E-40 | 2.93E-37 | 80.54288 |
| HSPB6 | 1.326151 | 1.731395 | 14.40298 | 4.00E-40 | 3.51E-37 | 80.34043 |
| SFTA1P | 1.861629 | 1.986882 | 14.37702 | 5.25E-40 | 4.50E-37 | 80.06994 |
| RGCC | 1.363947 | 4.872755 | 14.32089 | 9.46E-40 | 7.93E-37 | 79.48581 |
| RNASE1 | 1.561557 | 6.171828 | 14.30644 | 1.10E-39 | 9.04E-37 | 79.33554 |
| DLC1 | 1.268564 | 1.779388 | 14.27785 | 1.48E-39 | 1.17E-36 | 79.03859 |
| DRAM1 | 1.169302 | 3.613752 | 14.23625 | 2.29E-39 | 1.73E-36 | 78.60697 |
| SOD3 | 1.243738 | 3.046577 | 14.21036 | 3.00E-39 | 2.15E-36 | 78.33856 |
| SCN7A | 1.004811 | 0.779463 | 14.18414 | 3.95E-39 | 2.77E-36 | 78.06707 |
| LMOD1 | 1.100601 | 1.885638 | 14.17439 | 4.37E-39 | 2.96E-36 | 77.96611 |
| PGC | 2.469421 | 2.273883 | 14.14179 | 6.14E-39 | 4.02E-36 | 77.62889 |
| ELN | 1.522614 | 2.868798 | 14.10026 | 9.46E-39 | 5.89E-36 | 77.1999 |
| FBLN5 | 1.222522 | 2.534485 | 14.09622 | 9.87E-39 | 6.05E-36 | 77.15815 |
| LPL | 1.531985 | 2.049506 | 14.04668 | 1.65E-38 | 9.66E-36 | 76.64722 |
| EMCN | 1.026609 | 1.249866 | 14.02206 | 2.13E-38 | 1.19E-35 | 76.39362 |
| ATOH8 | 1.069086 | 0.983133 | 14.02053 | 2.17E-38 | 1.19E-35 | 76.37789 |
| GNG11 | 1.174178 | 2.557067 | 14.01012 | 2.41E-38 | 1.29E-35 | 76.27071 |
| S1PR1 | 1.308142 | 2.624222 | 13.97973 | 3.31E-38 | 1.75E-35 | 75.95809 |
| SUSD2 | 1.835116 | 2.478751 | 13.97386 | 3.51E-38 | 1.83E-35 | 75.89777 |
| C8orf34-AS1 | 1.099714 | 1.007113 | 13.92309 | 5.94E-38 | 3.02E-35 | 75.37626 |
| RHOBTB2 | 1.17543 | 2.395541 | 13.88654 | 8.67E-38 | 4.35E-35 | 75.00139 |
| NRGN | 1.491331 | 2.909869 | 13.88301 | 8.99E-38 | 4.45E-35 | 74.96524 |
| COLEC12 | 1.163806 | 1.947046 | 13.84714 | 1.30E-37 | 6.28E-35 | 74.59787 |
| TCIM | 1.62143 | 5.099085 | 13.81008 | 1.91E-37 | 8.76E-35 | 74.21876 |
| GJA5 | 1.193447 | 2.536863 | 13.74771 | 3.62E-37 | 1.61E-34 | 73.58184 |
| NKX2-1 | 1.73257 | 1.925052 | 13.73877 | 3.97E-37 | 1.72E-34 | 73.4907 |
| CLEC3B | 1.606846 | 2.242613 | 13.73768 | 4.02E-37 | 1.72E-34 | 73.47964 |
| SFTPB | 3.555842 | 6.705406 | 13.73529 | 4.12E-37 | 1.75E-34 | 73.45527 |
| EDNRB | 1.286463 | 1.595216 | 13.72891 | 4.40E-37 | 1.82E-34 | 73.39021 |
| CYP4B1 | 2.129374 | 2.339261 | 13.72308 | 4.67E-37 | 1.92E-34 | 73.33076 |
| C1orf162 | 1.0693 | 2.405687 | 13.7116 | 5.25E-37 | 2.11E-34 | 73.21377 |
| DNALI1 | 1.047352 | 1.227703 | 13.70599 | 5.56E-37 | 2.21E-34 | 73.15663 |
| CDH5 | 1.210662 | 2.589062 | 13.69621 | 6.15E-37 | 2.42E-34 | 73.05701 |
| ACVRL1 | 1.07835 | 2.583414 | 13.68521 | 6.89E-37 | 2.68E-34 | 72.94508 |
| AGER | 2.515988 | 3.384825 | 13.63242 | 1.18E-36 | 4.43E-34 | 72.40836 |
| CTSE | 2.161515 | 3.187854 | 13.59886 | 1.67E-36 | 6.01E-34 | 72.06761 |
| SFTPA1 | 3.883012 | 6.044672 | 13.59679 | 1.70E-36 | 6.09E-34 | 72.04658 |
| TGFBR2 | 1.203832 | 4.221677 | 13.54812 | 2.80E-36 | 9.65E-34 | 71.55325 |
| CACNA2D2 | 1.49285 | 1.534674 | 13.52959 | 3.38E-36 | 1.15E-33 | 71.36574 |
| LY86 | 1.095853 | 2.33927 | 13.52801 | 3.44E-36 | 1.15E-33 | 71.34966 |
| PLA2G1B | 1.360735 | 0.983966 | 13.52794 | 3.44E-36 | 1.15E-33 | 71.34896 |
| ABCA3 | 1.925986 | 3.076339 | 13.51932 | 3.76E-36 | 1.25E-33 | 71.2618 |
| CLDN18 | 2.275325 | 1.836402 | 13.46857 | 6.30E-36 | 1.96E-33 | 70.7489 |
| SFTPC | 3.992652 | 4.130078 | 13.46619 | 6.45E-36 | 1.99E-33 | 70.72487 |
| SLC22A31 | 1.523972 | 1.817051 | 13.46238 | 6.71E-36 | 2.05E-33 | 70.68641 |
| SLC39A8 | 1.431958 | 2.952663 | 13.4427 | 8.19E-36 | 2.43E-33 | 70.48781 |
| AGR3 | 1.864285 | 2.702449 | 13.43147 | 9.19E-36 | 2.71E-33 | 70.37453 |
| ADA2 | 1.184408 | 3.504753 | 13.43058 | 9.27E-36 | 2.71E-33 | 70.36554 |
| CITED2 | 1.105683 | 4.238271 | 13.42726 | 9.59E-36 | 2.76E-33 | 70.3321 |
| PODN | 1.100753 | 2.485127 | 13.42561 | 9.75E-36 | 2.79E-33 | 70.31541 |
| SFTPA2 | 3.827457 | 6.382199 | 13.37213 | 1.68E-35 | 4.66E-33 | 69.77682 |
| PTGDS | 1.639138 | 3.893078 | 13.36178 | 1.86E-35 | 5.14E-33 | 69.67269 |
| ARRB1 | 1.034602 | 1.877066 | 13.33852 | 2.36E-35 | 6.37E-33 | 69.4389 |
| CGNL1 | 1.035282 | 1.64327 | 13.33842 | 2.36E-35 | 6.37E-33 | 69.43793 |
| CELF2 | 1.020551 | 2.00487 | 13.32529 | 2.70E-35 | 7.23E-33 | 69.306 |
| HIGD1B | 1.040552 | 1.577274 | 13.28803 | 3.93E-35 | 1.02E-32 | 68.93205 |
| CYP2B7P | 1.640124 | 1.732307 | 13.19564 | 9.99E-35 | 2.47E-32 | 68.00716 |
| FGR | 1.056647 | 2.55492 | 13.19234 | 1.03E-34 | 2.54E-32 | 67.97416 |
| PPP1R14A | 1.005035 | 2.024249 | 13.17675 | 1.21E-34 | 2.95E-32 | 67.81847 |
| CADM1 | 1.186576 | 2.080598 | 13.17147 | 1.27E-34 | 3.09E-32 | 67.76573 |
| TMEM100 | 1.474085 | 1.188113 | 13.16356 | 1.38E-34 | 3.30E-32 | 67.6868 |
| PLN | 1.031234 | 1.534491 | 13.15945 | 1.44E-34 | 3.40E-32 | 67.64577 |
| DES | 1.51537 | 1.604048 | 13.12976 | 1.94E-34 | 4.53E-32 | 67.34964 |
| ROS1 | 1.324761 | 1.659757 | 13.11891 | 2.16E-34 | 5.02E-32 | 67.24159 |
| ENPP2 | 1.053619 | 2.63895 | 13.09519 | 2.74E-34 | 6.30E-32 | 67.00541 |
| ALOX5 | 1.262501 | 2.988979 | 13.08358 | 3.08E-34 | 7.04E-32 | 66.88979 |
| TGM2 | 1.406835 | 4.673407 | 13.06643 | 3.66E-34 | 8.31E-32 | 66.71926 |
| CYP27A1 | 1.309674 | 3.638548 | 13.04582 | 4.50E-34 | 9.92E-32 | 66.51446 |
| METTL7A | 1.237636 | 3.895964 | 12.99391 | 7.56E-34 | 1.64E-31 | 65.99938 |
| SERPINA1 | 1.841584 | 5.112917 | 12.98784 | 8.03E-34 | 1.73E-31 | 65.93921 |
| RPS29P11 | 1.064136 | 0.949469 | 12.97502 | 9.13E-34 | 1.95E-31 | 65.81216 |
| CFI | 1.073315 | 2.916392 | 12.95711 | 1.09E-33 | 2.32E-31 | 65.63483 |
| ENG | 1.02514 | 4.575066 | 12.94614 | 1.22E-33 | 2.54E-31 | 65.52622 |
| DEPP1 | 1.279327 | 4.619561 | 12.94605 | 1.22E-33 | 2.54E-31 | 65.52536 |
| AGTR2 | 1.142158 | 0.823826 | 12.92593 | 1.49E-33 | 3.03E-31 | 65.32643 |
| ALPL | 1.561307 | 3.810564 | 12.91705 | 1.63E-33 | 3.29E-31 | 65.23861 |
| MS4A7 | 1.195223 | 2.529659 | 12.91574 | 1.65E-33 | 3.32E-31 | 65.22567 |
| DPYSL2 | 1.106736 | 3.414281 | 12.91223 | 1.71E-33 | 3.42E-31 | 65.19098 |
| NPR1 | 1.025887 | 1.306027 | 12.89438 | 2.04E-33 | 4.06E-31 | 65.01474 |
| PTGIS | 1.065117 | 2.062401 | 12.86664 | 2.69E-33 | 5.27E-31 | 64.741 |
| GKN2 | 1.360944 | 0.882855 | 12.83504 | 3.68E-33 | 7.10E-31 | 64.4296 |
| HOPX | 1.345797 | 2.746785 | 12.82474 | 4.08E-33 | 7.83E-31 | 64.32825 |
| SLC44A4 | 1.426802 | 2.30958 | 12.82152 | 4.21E-33 | 8.04E-31 | 64.29652 |
| ACSL5 | 1.2235 | 2.661179 | 12.80702 | 4.86E-33 | 9.11E-31 | 64.15384 |
| FLRT3 | 1.313724 | 2.133092 | 12.80257 | 5.08E-33 | 9.40E-31 | 64.11003 |
| CHRDL1 | 1.349084 | 1.622726 | 12.79802 | 5.31E-33 | 9.77E-31 | 64.06536 |
| FBP1 | 1.5641 | 3.980622 | 12.79224 | 5.63E-33 | 1.03E-30 | 64.00847 |
| CD93 | 1.17662 | 3.222664 | 12.77396 | 6.75E-33 | 1.22E-30 | 63.8289 |
| CTSH | 1.215355 | 5.090029 | 12.7287 | 1.06E-32 | 1.85E-30 | 63.38474 |
| CPA3 | 1.383239 | 3.167089 | 12.72861 | 1.06E-32 | 1.85E-30 | 63.38385 |
| CLIC5 | 1.381074 | 1.211285 | 12.71849 | 1.17E-32 | 2.02E-30 | 63.28466 |
| SPON1 | 1.188536 | 2.823088 | 12.71525 | 1.21E-32 | 2.08E-30 | 63.25286 |
| C2 | 1.023482 | 2.844369 | 12.69388 | 1.49E-32 | 2.53E-30 | 63.04354 |
| GPIHBP1 | 1.235352 | 0.970327 | 12.68334 | 1.65E-32 | 2.77E-30 | 62.94038 |
| GMFG | 1.04803 | 3.448486 | 12.67933 | 1.72E-32 | 2.86E-30 | 62.90119 |
| GGTLC1 | 1.3334 | 0.863086 | 12.67168 | 1.85E-32 | 3.07E-30 | 62.82638 |
| RASSF2 | 1.021129 | 2.297519 | 12.65772 | 2.13E-32 | 3.49E-30 | 62.68989 |
| LRRC32 | 1.071444 | 2.889718 | 12.64358 | 2.45E-32 | 4.00E-30 | 62.55173 |
| CSRNP1 | 1.121603 | 3.363587 | 12.63972 | 2.54E-32 | 4.12E-30 | 62.51397 |
| GIMAP6 | 1.03555 | 2.227197 | 12.63197 | 2.74E-32 | 4.43E-30 | 62.43834 |
| ITM2A | 1.155171 | 2.769107 | 12.62734 | 2.87E-32 | 4.61E-30 | 62.39309 |
| ACTG2 | 1.075659 | 2.124136 | 12.61583 | 3.21E-32 | 5.13E-30 | 62.28079 |
| FAM107A | 1.444517 | 1.31032 | 12.61262 | 3.32E-32 | 5.27E-30 | 62.24946 |
| ALOX5AP | 1.3292 | 3.663677 | 12.59875 | 3.80E-32 | 5.96E-30 | 62.11418 |
| FPR1 | 1.166392 | 2.367108 | 12.5946 | 3.96E-32 | 6.19E-30 | 62.07377 |
| PAPSS2 | 1.206923 | 3.401751 | 12.5526 | 5.98E-32 | 9.20E-30 | 61.66478 |
| ACKR1 | 1.442428 | 2.106436 | 12.5358 | 7.05E-32 | 1.08E-29 | 61.5013 |
| MYL9 | 1.131535 | 5.639084 | 12.53313 | 7.24E-32 | 1.10E-29 | 61.47537 |
| MSR1 | 1.243361 | 2.415009 | 12.5304 | 7.44E-32 | 1.13E-29 | 61.44885 |
| PELATON | 1.019296 | 1.936095 | 12.51795 | 8.40E-32 | 1.26E-29 | 61.32782 |
| TNNC1 | 1.511504 | 1.478935 | 12.49321 | 1.07E-31 | 1.57E-29 | 61.08764 |
| SLIT3 | 1.009066 | 1.391767 | 12.44794 | 1.67E-31 | 2.39E-29 | 60.64882 |
| GADD45B | 1.077705 | 4.225046 | 12.39103 | 2.90E-31 | 4.08E-29 | 60.0983 |
| DMBT1 | 1.88795 | 2.283331 | 12.38067 | 3.21E-31 | 4.48E-29 | 59.99825 |
| LYVE1 | 1.043259 | 1.367608 | 12.34268 | 4.65E-31 | 6.33E-29 | 59.63178 |
| MCEMP1 | 1.558317 | 1.551765 | 12.33901 | 4.82E-31 | 6.52E-29 | 59.59646 |
| HSD17B6 | 1.131141 | 1.841544 | 12.3051 | 6.70E-31 | 8.98E-29 | 59.26994 |
| HLA-DOA | 1.203793 | 3.086614 | 12.2442 | 1.21E-30 | 1.57E-28 | 58.68471 |
| EVI2B | 1.114524 | 3.046139 | 12.20912 | 1.70E-30 | 2.18E-28 | 58.34846 |
| SLCO2B1 | 1.126854 | 2.537783 | 12.20103 | 1.83E-30 | 2.34E-28 | 58.27097 |
| SCNN1B | 1.265847 | 2.478652 | 12.18519 | 2.14E-30 | 2.70E-28 | 58.1193 |
| CPM | 1.241543 | 2.539422 | 12.17746 | 2.30E-30 | 2.89E-28 | 58.0454 |
| PIGR | 2.042718 | 3.455799 | 12.13908 | 3.34E-30 | 4.11E-28 | 57.6786 |
| TMC5 | 1.147738 | 1.774859 | 12.13058 | 3.62E-30 | 4.45E-28 | 57.59744 |
| FCN3 | 1.593003 | 1.946585 | 12.06545 | 6.77E-30 | 8.02E-28 | 56.97684 |
| FMO2 | 1.379321 | 2.335348 | 12.05015 | 7.84E-30 | 9.23E-28 | 56.83132 |
| C5AR1 | 1.028502 | 3.046986 | 12.02747 | 9.75E-30 | 1.12E-27 | 56.6158 |
| KLF2 | 1.087519 | 2.652932 | 12.01246 | 1.13E-29 | 1.27E-27 | 56.47331 |
| COL8A1 | 1.094056 | 2.959866 | 12.00813 | 1.17E-29 | 1.32E-27 | 56.4322 |
| VSIG4 | 1.487415 | 3.607785 | 12.00811 | 1.17E-29 | 1.32E-27 | 56.43208 |
| PRR15L | 1.278083 | 2.464662 | 11.98724 | 1.43E-29 | 1.58E-27 | 56.23418 |
| CD52 | 1.417828 | 4.540792 | 11.94529 | 2.14E-29 | 2.32E-27 | 55.83692 |
| PLIN2 | 1.013157 | 3.403259 | 11.94159 | 2.22E-29 | 2.39E-27 | 55.80189 |
| CD4 | 1.046806 | 3.949224 | 11.90863 | 3.03E-29 | 3.22E-27 | 55.49047 |
| MRC1 | 1.474881 | 2.737163 | 11.86437 | 4.62E-29 | 4.80E-27 | 55.07301 |
| SELPLG | 1.056664 | 3.385431 | 11.85899 | 4.87E-29 | 5.01E-27 | 55.02238 |
| TYROBP | 1.173866 | 5.711153 | 11.8489 | 5.36E-29 | 5.48E-27 | 54.92739 |
| KRT7 | 2.069179 | 4.174386 | 11.84147 | 5.75E-29 | 5.87E-27 | 54.85744 |
| MNDA | 1.062598 | 2.579463 | 11.82393 | 6.79E-29 | 6.88E-27 | 54.69245 |
| FOXA2 | 1.129029 | 1.205028 | 11.81727 | 7.23E-29 | 7.26E-27 | 54.62978 |
| GIMAP7 | 1.075137 | 2.833881 | 11.79443 | 8.98E-29 | 8.89E-27 | 54.41527 |
| TMPRSS2 | 1.284214 | 2.139494 | 11.7868 | 9.66E-29 | 9.51E-27 | 54.3436 |
| FAM189A2 | 1.034096 | 1.183095 | 11.73321 | 1.60E-28 | 1.53E-26 | 53.84138 |
| MS4A4A | 1.043675 | 2.725149 | 11.72576 | 1.72E-28 | 1.63E-26 | 53.77168 |
| SCGB3A2 | 2.444676 | 4.246667 | 11.72298 | 1.77E-28 | 1.67E-26 | 53.74571 |
| FOLR2 | 1.094757 | 3.074932 | 11.70869 | 2.02E-28 | 1.90E-26 | 53.61204 |
| RAMP2 | 1.000899 | 3.401953 | 11.6711 | 2.88E-28 | 2.65E-26 | 53.26101 |
| HLA-DMA | 1.106051 | 4.659902 | 11.64899 | 3.55E-28 | 3.22E-26 | 53.05486 |
| KIT | 1.249231 | 1.931332 | 11.64835 | 3.57E-28 | 3.22E-26 | 53.0489 |
| C11orf96 | 1.167979 | 2.946391 | 11.63471 | 4.06E-28 | 3.61E-26 | 52.92181 |
| CXCL2 | 1.625105 | 3.111071 | 11.63296 | 4.12E-28 | 3.67E-26 | 52.9055 |
| TAGLN | 1.05831 | 5.203395 | 11.60013 | 5.61E-28 | 4.88E-26 | 52.60014 |
| SERPING1 | 1.026699 | 5.98596 | 11.57797 | 6.91E-28 | 5.96E-26 | 52.39423 |
| RND1 | 1.247395 | 1.862139 | 11.54864 | 9.09E-28 | 7.80E-26 | 52.12218 |
| MAMDC2 | 1.09614 | 1.178637 | 11.5466 | 9.27E-28 | 7.91E-26 | 52.1032 |
| FGG | 1.821406 | 1.907714 | 11.52387 | 1.15E-27 | 9.66E-26 | 51.89267 |
| CTSS | 1.154098 | 4.853448 | 11.51987 | 1.19E-27 | 9.96E-26 | 51.85558 |
| CLDN5 | 1.118608 | 2.510973 | 11.4926 | 1.53E-27 | 1.28E-25 | 51.60341 |
| CA4 | 1.039837 | 0.715345 | 11.47719 | 1.77E-27 | 1.47E-25 | 51.46105 |
| C3 | 1.290146 | 5.407856 | 11.45964 | 2.09E-27 | 1.71E-25 | 51.299 |
| STEAP4 | 1.249729 | 1.811358 | 11.45554 | 2.17E-27 | 1.77E-25 | 51.26121 |
| TENT5C | 1.03184 | 2.542727 | 11.42096 | 2.99E-27 | 2.40E-25 | 50.9425 |
| TMEM119 | 1.021196 | 2.821545 | 11.41432 | 3.18E-27 | 2.52E-25 | 50.88137 |
| IL33 | 1.275102 | 2.751974 | 11.41166 | 3.26E-27 | 2.58E-25 | 50.85686 |
| COL14A1 | 1.086564 | 2.031826 | 11.40229 | 3.56E-27 | 2.78E-25 | 50.77068 |
| SAPCD2 | -1.03794 | 2.717203 | -11.3898 | 4.00E-27 | 3.10E-25 | 50.65587 |
| VEGFD | 1.154091 | 1.058648 | 11.37768 | 4.47E-27 | 3.43E-25 | 50.54446 |
| GIMAP4 | 1.000366 | 3.255882 | 11.37014 | 4.79E-27 | 3.66E-25 | 50.47519 |
| CD36 | 1.050212 | 1.702857 | 11.32736 | 7.13E-27 | 5.32E-25 | 50.08288 |
| SCGB3A1 | 2.255793 | 3.574653 | 11.30002 | 9.17E-27 | 6.71E-25 | 49.83261 |
| HLA-DPB1 | 1.185431 | 6.019173 | 11.28931 | 1.01E-26 | 7.35E-25 | 49.73462 |
| ACTA2 | 1.066658 | 5.437118 | 11.27892 | 1.11E-26 | 8.04E-25 | 49.63963 |
| LAPTM5 | 1.099556 | 5.908488 | 11.26401 | 1.28E-26 | 9.14E-25 | 49.50351 |
| RETN | 1.144191 | 1.081881 | 11.25175 | 1.43E-26 | 1.02E-24 | 49.39163 |
| ALDH3B1 | 1.057683 | 1.994843 | 11.24492 | 1.53E-26 | 1.08E-24 | 49.32935 |
| SRGN | 1.216984 | 6.185179 | 11.24142 | 1.58E-26 | 1.11E-24 | 49.29741 |
| VIM | 1.009708 | 6.594718 | 11.23433 | 1.68E-26 | 1.18E-24 | 49.2328 |
| CD53 | 1.10208 | 4.487314 | 11.2214 | 1.89E-26 | 1.31E-24 | 49.11495 |
| CRIP2 | 1.078079 | 3.419099 | 11.20054 | 2.29E-26 | 1.56E-24 | 48.92514 |
| PRELP | 1.236347 | 3.067815 | 11.19661 | 2.38E-26 | 1.62E-24 | 48.88937 |
| THBS1 | 1.092491 | 4.73875 | 11.19522 | 2.41E-26 | 1.64E-24 | 48.87676 |
| MARCO | 1.739746 | 3.209809 | 11.18749 | 2.59E-26 | 1.74E-24 | 48.80644 |
| SPI1 | 1.002067 | 3.79023 | 11.14953 | 3.66E-26 | 2.40E-24 | 48.46175 |
| VWF | 1.120866 | 3.819412 | 11.11888 | 4.85E-26 | 3.15E-24 | 48.18395 |
| CPB2 | 1.029614 | 0.655138 | 11.09873 | 5.83E-26 | 3.74E-24 | 48.00159 |
| LPCAT1 | 1.049454 | 4.567127 | 11.06307 | 8.07E-26 | 5.10E-24 | 47.67939 |
| HPN | 1.073087 | 1.663164 | 10.98485 | 1.64E-25 | 9.97E-24 | 46.97492 |
| PARM1 | 1.23215 | 3.247919 | 10.97082 | 1.87E-25 | 1.13E-23 | 46.8489 |
| SELENOP | 1.177988 | 3.892159 | 10.96245 | 2.02E-25 | 1.20E-23 | 46.7738 |
| ANKRD1 | 1.261589 | 1.010601 | 10.92535 | 2.82E-25 | 1.65E-23 | 46.44119 |
| RARRES2 | 1.073243 | 4.868216 | 10.92523 | 2.82E-25 | 1.65E-23 | 46.44014 |
| CD163 | 1.172989 | 3.313979 | 10.82332 | 7.08E-25 | 4.00E-23 | 45.53032 |
| CHI3L2 | 1.123342 | 1.64672 | 10.82222 | 7.15E-25 | 4.03E-23 | 45.5205 |
| CCN1 | 1.118714 | 5.459988 | 10.81043 | 7.95E-25 | 4.47E-23 | 45.41558 |
| NNMT | 1.050752 | 4.570814 | 10.79723 | 8.95E-25 | 4.98E-23 | 45.29824 |
| SLCO2A1 | 1.164073 | 2.685343 | 10.79447 | 9.17E-25 | 5.09E-23 | 45.27376 |
| CD74 | 1.172651 | 8.923456 | 10.78897 | 9.64E-25 | 5.34E-23 | 45.22492 |
| MOXD1 | 1.052874 | 2.651283 | 10.77884 | 1.06E-24 | 5.77E-23 | 45.13495 |
| ANOS1 | 1.006219 | 2.135785 | 10.76052 | 1.24E-24 | 6.72E-23 | 44.97239 |
| SPOCK2 | 1.302089 | 3.050905 | 10.71139 | 1.93E-24 | 1.02E-22 | 44.53743 |
| HLA-DMB | 1.023981 | 3.330913 | 10.68864 | 2.37E-24 | 1.23E-22 | 44.33644 |
| MYADM | 1.119981 | 4.747905 | 10.66135 | 3.02E-24 | 1.54E-22 | 44.0957 |
| EFEMP1 | 1.179282 | 4.34296 | 10.64085 | 3.62E-24 | 1.83E-22 | 43.91517 |
| MLPH | 1.123327 | 1.99057 | 10.63769 | 3.72E-24 | 1.87E-22 | 43.88733 |
| HLA-DPA1 | 1.203447 | 5.211937 | 10.61188 | 4.69E-24 | 2.32E-22 | 43.66033 |
| GPX3 | 1.319092 | 4.894629 | 10.61162 | 4.70E-24 | 2.32E-22 | 43.6581 |
| OLR1 | 1.275809 | 2.603496 | 10.54626 | 8.38E-24 | 4.03E-22 | 43.08496 |
| CYBB | 1.150368 | 3.693639 | 10.53563 | 9.21E-24 | 4.38E-22 | 42.9919 |
| APOC1 | 1.278622 | 5.30192 | 10.53199 | 9.51E-24 | 4.50E-22 | 42.96013 |
| ORM1 | 1.007607 | 1.240945 | 10.52404 | 1.02E-23 | 4.78E-22 | 42.89062 |
| TPSAB1 | 1.055829 | 3.102048 | 10.50241 | 1.23E-23 | 5.72E-22 | 42.70171 |
| TREM1 | 1.036003 | 2.023699 | 10.46306 | 1.75E-23 | 8.00E-22 | 42.35876 |
| FOXM1 | -1.04941 | 3.892306 | -10.4597 | 1.80E-23 | 8.22E-22 | 42.3292 |
| TPPP3 | 1.223488 | 3.103569 | 10.42864 | 2.36E-23 | 1.06E-21 | 42.05943 |
| RAMP3 | 1.02299 | 2.975392 | 10.4193 | 2.57E-23 | 1.15E-21 | 41.97837 |
| FOXE1 | -1.68477 | 3.181788 | -10.3442 | 4.95E-23 | 2.16E-21 | 41.32763 |
| ALOX15B | 1.190698 | 2.267703 | 10.33094 | 5.56E-23 | 2.42E-21 | 41.21352 |
| DCN | 1.168229 | 4.802551 | 10.30539 | 6.95E-23 | 2.99E-21 | 40.99313 |
| SLC7A5 | -1.20822 | 5.521928 | -10.2837 | 8.39E-23 | 3.59E-21 | 40.80656 |
| HLA-DRA | 1.250107 | 8.914802 | 10.28042 | 8.64E-23 | 3.68E-21 | 40.77815 |
| PSAT1 | -1.24404 | 4.667101 | -10.2739 | 9.14E-23 | 3.88E-21 | 40.72237 |
| CFD | 1.04454 | 3.558859 | 10.26473 | 9.90E-23 | 4.20E-21 | 40.6433 |
| TPX2 | -1.08833 | 4.708642 | -10.2552 | 1.08E-22 | 4.54E-21 | 40.56149 |
| RRAD | 1.25619 | 2.557895 | 10.24028 | 1.22E-22 | 5.14E-21 | 40.43329 |
| TOP2A | -1.08365 | 4.503865 | -10.2059 | 1.65E-22 | 6.75E-21 | 40.13864 |
| MS4A15 | 1.180583 | 1.007185 | 10.19655 | 1.79E-22 | 7.28E-21 | 40.05869 |
| MELTF | -1.0141 | 2.523782 | -10.1779 | 2.10E-22 | 8.49E-21 | 39.89962 |
| JCHAIN | 1.708694 | 6.731802 | 10.17459 | 2.16E-22 | 8.73E-21 | 39.87089 |
| TPSB2 | 1.131364 | 2.982064 | 10.14809 | 2.72E-22 | 1.08E-20 | 39.64477 |
| HLA-DRB1 | 1.179938 | 7.990009 | 10.12286 | 3.38E-22 | 1.33E-20 | 39.42981 |
| HBB | 1.783902 | 3.645022 | 10.0381 | 6.99E-22 | 2.67E-20 | 38.71029 |
| MUC1 | 1.274862 | 4.580434 | 9.98533 | 1.10E-21 | 4.08E-20 | 38.26447 |
| HSPB8 | 1.05959 | 3.210509 | 9.96788 | 1.27E-21 | 4.68E-20 | 38.1174 |
| GPRC5A | 1.337374 | 4.056072 | 9.89637 | 2.34E-21 | 8.23E-20 | 37.51657 |
| FOSB | 1.491605 | 3.018823 | 9.842723 | 3.69E-21 | 1.26E-19 | 37.0678 |
| NMRAL2P | -1.93905 | 2.857474 | -9.80487 | 5.07E-21 | 1.72E-19 | 36.75222 |
| ICAM1 | 1.155985 | 4.821481 | 9.801922 | 5.20E-21 | 1.75E-19 | 36.72764 |
| FOXJ1 | 1.188913 | 1.635618 | 9.777833 | 6.37E-21 | 2.13E-19 | 36.52726 |
| EPAS1 | 1.032948 | 5.058096 | 9.764997 | 7.09E-21 | 2.37E-19 | 36.42064 |
| FHL1 | 1.105221 | 2.377105 | 9.724035 | 1.00E-20 | 3.28E-19 | 36.08103 |
| HLA-DRB5 | 1.267305 | 6.201632 | 9.694795 | 1.28E-20 | 4.13E-19 | 35.83923 |
| CCL21 | 1.269761 | 4.835409 | 9.673305 | 1.53E-20 | 4.89E-19 | 35.66183 |
| SCGB1A1 | 2.502573 | 4.313718 | 9.651005 | 1.84E-20 | 5.83E-19 | 35.47805 |
| DPT | 1.106995 | 3.155851 | 9.649184 | 1.87E-20 | 5.91E-19 | 35.46306 |
| DUSP1 | 1.05573 | 6.183957 | 9.64271 | 1.97E-20 | 6.22E-19 | 35.40977 |
| FCGR3A | 1.039352 | 4.444777 | 9.634728 | 2.11E-20 | 6.61E-19 | 35.3441 |
| SLC7A11 | -1.276 | 2.323548 | -9.63154 | 2.17E-20 | 6.79E-19 | 35.31791 |
| PERP | -1.01691 | 7.825739 | -9.59257 | 3.00E-20 | 9.26E-19 | 34.99787 |
| SDR16C5 | 1.074659 | 2.554761 | 9.589281 | 3.08E-20 | 9.50E-19 | 34.97094 |
| RAPGEFL1 | -1.13043 | 3.37668 | -9.54993 | 4.27E-20 | 1.29E-18 | 34.64881 |
| C1QB | 1.140196 | 6.489229 | 9.519467 | 5.49E-20 | 1.63E-18 | 34.40016 |
| TRIM16L | -1.20965 | 2.751504 | -9.50857 | 6.00E-20 | 1.78E-18 | 34.31133 |
| C1QA | 1.075234 | 6.536519 | 9.4912 | 6.93E-20 | 2.03E-18 | 34.1699 |
| HPGD | 1.03373 | 1.978722 | 9.455668 | 9.28E-20 | 2.69E-18 | 33.88116 |
| CYP4F11 | -1.83363 | 2.807387 | -9.42281 | 1.22E-19 | 3.47E-18 | 33.61483 |
| LAMP3 | 1.129963 | 4.412827 | 9.391872 | 1.57E-19 | 4.43E-18 | 33.36469 |
| TP63 | -1.58726 | 5.129164 | -9.29543 | 3.44E-19 | 9.33E-18 | 32.58872 |
| CCL2 | 1.03146 | 4.111968 | 9.270841 | 4.20E-19 | 1.13E-17 | 32.39184 |
| UBE2C | -1.07293 | 5.162107 | -9.26533 | 4.39E-19 | 1.18E-17 | 32.34779 |
| C1QC | 1.029324 | 6.52994 | 9.258223 | 4.65E-19 | 1.24E-17 | 32.29095 |
| KRT6A | -2.49198 | 8.395173 | -9.2455 | 5.15E-19 | 1.37E-17 | 32.18932 |
| CCN2 | 1.01724 | 5.747687 | 9.241184 | 5.34E-19 | 1.41E-17 | 32.15486 |
| CXCL17 | 1.462351 | 5.115595 | 9.211766 | 6.77E-19 | 1.77E-17 | 31.92034 |
| RBP4 | 1.006284 | 1.399337 | 9.17155 | 9.36E-19 | 2.40E-17 | 31.60061 |
| CAVIN2 | 1.192182 | 3.119081 | 9.16303 | 1.00E-18 | 2.57E-17 | 31.53301 |
| HBA2 | 1.282006 | 2.300487 | 9.149318 | 1.12E-18 | 2.85E-17 | 31.4243 |
| HLA-DRB6 | 1.114883 | 4.005715 | 9.139473 | 1.21E-18 | 3.07E-17 | 31.34633 |
| ITGB6 | 1.01192 | 3.701833 | 9.092538 | 1.76E-18 | 4.39E-17 | 30.97544 |
| CLIC3 | 1.034668 | 2.765779 | 8.980988 | 4.28E-18 | 1.02E-16 | 30.09962 |
| LYZ | 1.371825 | 6.560563 | 8.978259 | 4.38E-18 | 1.04E-16 | 30.07829 |
| HLA-DQA1 | 1.048134 | 4.151773 | 8.946824 | 5.61E-18 | 1.32E-16 | 29.833 |
| SLC2A1 | -1.34697 | 6.880824 | -8.87943 | 9.55E-18 | 2.18E-16 | 29.30926 |
| HLA-DQB1 | 1.037917 | 4.546917 | 8.848827 | 1.21E-17 | 2.75E-16 | 29.0724 |
| MYBL2 | -1.00506 | 4.750111 | -8.83432 | 1.36E-17 | 3.07E-16 | 28.96032 |
| CBR3 | -1.04971 | 3.44826 | -8.81608 | 1.57E-17 | 3.52E-16 | 28.81962 |
| SLC6A14 | 1.039325 | 2.310166 | 8.790522 | 1.92E-17 | 4.26E-16 | 28.62285 |
| SLC6A8 | -1.17227 | 5.176609 | -8.77132 | 2.23E-17 | 4.90E-16 | 28.47526 |
| HLA-DQB2 | 1.022505 | 3.215695 | 8.729829 | 3.07E-17 | 6.66E-16 | 28.15726 |
| SFN | -1.11367 | 8.562064 | -8.72115 | 3.29E-17 | 7.09E-16 | 28.09091 |
| CYP4F3 | -1.57584 | 2.19151 | -8.71769 | 3.38E-17 | 7.27E-16 | 28.06445 |
| AZGP1 | 1.174531 | 1.694234 | 8.642812 | 6.02E-17 | 1.27E-15 | 27.49399 |
| PTGR1 | -1.13213 | 4.177983 | -8.58998 | 9.04E-17 | 1.87E-15 | 27.09375 |
| PKP1 | -1.72037 | 5.975471 | -8.58625 | 9.30E-17 | 1.92E-15 | 27.06557 |
| HP | 1.109071 | 1.790809 | 8.579513 | 9.80E-17 | 2.02E-15 | 27.01468 |
| GBP6 | -1.44071 | 3.615692 | -8.57901 | 9.83E-17 | 2.03E-15 | 27.01089 |
| MSLN | 1.416447 | 2.616274 | 8.554819 | 1.18E-16 | 2.42E-15 | 26.82843 |
| FABP4 | 1.402522 | 1.947234 | 8.531487 | 1.41E-16 | 2.86E-15 | 26.65282 |
| FGFBP1 | -1.46774 | 4.78831 | -8.52255 | 1.51E-16 | 3.05E-15 | 26.58564 |
| DSP | -1.01899 | 6.230734 | -8.52196 | 1.52E-16 | 3.07E-15 | 26.58121 |
| SERPINB5 | -1.38857 | 4.760658 | -8.46564 | 2.33E-16 | 4.61E-15 | 26.1592 |
| ABCC5 | -1.03613 | 4.185566 | -8.40857 | 3.60E-16 | 6.91E-15 | 25.73379 |
| GCLC | -1.05462 | 4.194552 | -8.39971 | 3.85E-16 | 7.35E-15 | 25.66793 |
| AKR1B10 | -2.24841 | 4.974931 | -8.37773 | 4.54E-16 | 8.60E-15 | 25.50475 |
| COL7A1 | -1.25056 | 3.914685 | -8.37004 | 4.81E-16 | 9.10E-15 | 25.44779 |
| DSG3 | -1.66335 | 4.825609 | -8.28287 | 9.24E-16 | 1.69E-14 | 24.80457 |
| ADAM23 | -1.35939 | 2.852145 | -8.28224 | 9.29E-16 | 1.70E-14 | 24.79988 |
| SOX15 | -1.1509 | 3.365747 | -8.23844 | 1.29E-15 | 2.30E-14 | 24.47874 |
| CCL18 | 1.186212 | 5.276685 | 8.223235 | 1.44E-15 | 2.56E-14 | 24.36751 |
| MIR205HG | -1.2386 | 4.115527 | -8.21543 | 1.53E-15 | 2.71E-14 | 24.31047 |
| PITX1 | -1.19512 | 3.798743 | -8.17075 | 2.13E-15 | 3.69E-14 | 23.9849 |
| CEACAM6 | 1.492475 | 4.787332 | 8.161807 | 2.27E-15 | 3.92E-14 | 23.91991 |
| DLX5 | -1.04763 | 2.618936 | -8.0962 | 3.68E-15 | 6.16E-14 | 23.44473 |
| GJB5 | -1.14306 | 3.898081 | -8.08661 | 3.95E-15 | 6.58E-14 | 23.37556 |
| WFDC2 | 1.214715 | 5.321603 | 8.084229 | 4.02E-15 | 6.68E-14 | 23.35838 |
| NECTIN1 | -1.05459 | 5.428906 | -8.03371 | 5.81E-15 | 9.43E-14 | 22.99495 |
| TNS4 | -1.13853 | 4.038926 | -7.98913 | 8.04E-15 | 1.28E-13 | 22.67579 |
| GPX2 | -1.91205 | 5.732011 | -7.97933 | 8.64E-15 | 1.37E-13 | 22.60584 |
| C20orf85 | 1.169785 | 1.42579 | 7.916716 | 1.36E-14 | 2.11E-13 | 22.16034 |
| GJB3 | -1.01932 | 3.66272 | -7.8324 | 2.49E-14 | 3.75E-13 | 21.56484 |
| KRT16 | -1.88958 | 6.046337 | -7.78576 | 3.47E-14 | 5.16E-13 | 21.2376 |
| HLA-DQA2 | 1.173216 | 3.723235 | 7.763071 | 4.08E-14 | 6.00E-13 | 21.07902 |
| FBN2 | -1.05738 | 1.57202 | -7.7361 | 4.94E-14 | 7.19E-13 | 20.89097 |
| UPK3B | 1.06702 | 2.183563 | 7.67931 | 7.39E-14 | 1.05E-12 | 20.49668 |
| GJB6 | -1.55315 | 4.042576 | -7.62652 | 1.07E-13 | 1.49E-12 | 20.13224 |
| ADH7 | -1.66731 | 3.418329 | -7.62588 | 1.08E-13 | 1.50E-12 | 20.12786 |
| KRT17 | -1.78886 | 8.460928 | -7.60602 | 1.24E-13 | 1.71E-12 | 19.99135 |
| KRT6B | -1.77852 | 5.297158 | -7.59228 | 1.36E-13 | 1.88E-12 | 19.89701 |
| TRIM29 | -1.11539 | 4.961511 | -7.56031 | 1.70E-13 | 2.32E-12 | 19.67809 |
| DSC3 | -1.32254 | 4.538776 | -7.50052 | 2.57E-13 | 3.44E-12 | 19.27075 |
| AKR1C1 | -1.58496 | 4.117002 | -7.48064 | 2.95E-13 | 3.92E-12 | 19.13591 |
| SOX2 | -1.40783 | 5.033654 | -7.39098 | 5.47E-13 | 7.04E-12 | 18.53124 |
| OSGIN1 | -1.07167 | 2.94696 | -7.37181 | 6.24E-13 | 7.97E-12 | 18.40276 |
| AKR1C2 | -1.57667 | 4.472845 | -7.35128 | 7.17E-13 | 9.10E-12 | 18.26545 |
| ALDH3A1 | -1.62707 | 4.187037 | -7.18404 | 2.22E-12 | 2.67E-11 | 17.1586 |
| UGT1A7 | -1.03229 | 1.268799 | -7.12846 | 3.21E-12 | 3.79E-11 | 16.79542 |
| NTRK2 | -1.16319 | 2.998054 | -6.97094 | 9.07E-12 | 1.00E-10 | 15.77892 |
| GPR87 | -1.08699 | 4.305826 | -6.958 | 9.87E-12 | 1.09E-10 | 15.69626 |
| KRT5 | -1.84078 | 8.603167 | -6.87224 | 1.72E-11 | 1.84E-10 | 15.15164 |
| AKR1C3 | -1.38068 | 4.546224 | -6.85916 | 1.87E-11 | 1.99E-10 | 15.0691 |
| SOST | -1.1452 | 1.463773 | -6.76473 | 3.43E-11 | 3.53E-10 | 14.47702 |
| PTHLH | -1.31057 | 4.235311 | -6.75323 | 3.69E-11 | 3.78E-10 | 14.40539 |
| CLCA2 | -1.41226 | 4.576102 | -6.73079 | 4.26E-11 | 4.33E-10 | 14.26588 |
| LYPD3 | -1.11699 | 4.759709 | -6.62322 | 8.40E-11 | 8.24E-10 | 13.6027 |
| WIF1 | 1.173534 | 1.944638 | 6.607826 | 9.24E-11 | 9.03E-10 | 13.50855 |
| AQP3 | 1.108361 | 5.311331 | 6.59493 | 1.00E-10 | 9.74E-10 | 13.42981 |
| IGHA2 | 1.243437 | 6.680446 | 6.58224 | 1.08E-10 | 1.05E-09 | 13.35246 |
| IGLV2-18 | 1.006247 | 2.817458 | 6.557451 | 1.27E-10 | 1.21E-09 | 13.20173 |
| IGHA1 | 1.251922 | 10.06006 | 6.516673 | 1.63E-10 | 1.54E-09 | 12.95482 |
| UCHL1 | -1.21514 | 4.515435 | -6.41042 | 3.13E-10 | 2.85E-09 | 12.31769 |
| PRAME | -1.05658 | 3.183932 | -6.39985 | 3.34E-10 | 3.03E-09 | 12.25479 |
| CSTA | -1.03533 | 6.054286 | -6.37195 | 3.96E-10 | 3.56E-09 | 12.08921 |
| IGLV5-45 | 1.140183 | 3.313433 | 6.342959 | 4.72E-10 | 4.20E-09 | 11.91784 |
| LINC01133 | -1.02257 | 2.63694 | -6.33844 | 4.85E-10 | 4.31E-09 | 11.89118 |
| NQO1 | -1.03741 | 5.028039 | -6.32383 | 5.29E-10 | 4.68E-09 | 11.80512 |
| GJB2 | -1.20951 | 5.202359 | -6.31129 | 5.71E-10 | 5.03E-09 | 11.7314 |
| IGLV2-23 | 1.187454 | 6.515059 | 6.273463 | 7.17E-10 | 6.22E-09 | 11.50975 |
| IGHD | 1.239117 | 3.489803 | 6.212467 | 1.03E-09 | 8.76E-09 | 11.15479 |
| IGLV2-8 | 1.128881 | 4.814656 | 6.188676 | 1.19E-09 | 1.00E-08 | 11.01716 |
| IGLV3-19 | 1.227327 | 6.731993 | 6.15128 | 1.48E-09 | 1.23E-08 | 10.80174 |
| KRT14 | -1.78999 | 5.38689 | -6.06866 | 2.41E-09 | 1.93E-08 | 10.32984 |
| IGLV2-11 | 1.182537 | 6.08641 | 6.01076 | 3.37E-09 | 2.65E-08 | 10.00248 |
| BPIFB1 | 1.267212 | 2.719226 | 5.983284 | 3.95E-09 | 3.07E-08 | 9.848086 |
| IGHV4-59 | 1.132994 | 5.628612 | 5.931683 | 5.31E-09 | 4.05E-08 | 9.559816 |
| LTF | 1.092847 | 3.764926 | 5.889491 | 6.76E-09 | 5.08E-08 | 9.325738 |
| IGLV2-14 | 1.140796 | 7.092908 | 5.843744 | 8.76E-09 | 6.48E-08 | 9.073596 |
| IGLV3-10 | 1.192795 | 5.251383 | 5.813329 | 1.04E-08 | 7.62E-08 | 8.906922 |
| IGLV1-51 | 1.096062 | 6.539682 | 5.810676 | 1.06E-08 | 7.73E-08 | 8.89242 |
| IGLV3-27 | 1.03372 | 3.532206 | 5.711604 | 1.84E-08 | 1.30E-07 | 8.355068 |
| IGKV1-6 | 1.022196 | 4.653856 | 5.697476 | 1.99E-08 | 1.40E-07 | 8.279107 |
| SPRR1A | -1.57418 | 5.1336 | -5.68445 | 2.13E-08 | 1.50E-07 | 8.209209 |
| COL17A1 | -1.09386 | 4.262877 | -5.6792 | 2.20E-08 | 1.54E-07 | 8.181075 |
| CALML3 | -1.34618 | 5.133228 | -5.64682 | 2.63E-08 | 1.83E-07 | 8.008126 |
| IGHV3-15 | 1.058276 | 5.765198 | 5.641219 | 2.71E-08 | 1.88E-07 | 7.978292 |
| S100A2 | -1.30507 | 6.979461 | -5.62434 | 2.97E-08 | 2.05E-07 | 7.888559 |
| IGLV1-44 | 1.064911 | 6.180084 | 5.585279 | 3.68E-08 | 2.51E-07 | 7.681804 |
| IGKV1-27 | 1.040039 | 4.559598 | 5.578617 | 3.81E-08 | 2.60E-07 | 7.646673 |
| IGKV3-11 | 1.060578 | 7.24438 | 5.561657 | 4.18E-08 | 2.83E-07 | 7.557397 |
| IGKV2-24 | 1.008514 | 4.097963 | 5.536399 | 4.79E-08 | 3.22E-07 | 7.424899 |
| IGKV3-20 | 1.038576 | 7.933693 | 5.483766 | 6.36E-08 | 4.20E-07 | 7.150521 |
| SPRR1B | -1.52075 | 5.526969 | -5.47255 | 6.76E-08 | 4.45E-07 | 7.09237 |
| IGLV3-1 | 1.035623 | 5.847347 | 5.470096 | 6.84E-08 | 4.50E-07 | 7.079643 |
| IGLV4-69 | 1.031281 | 5.006441 | 5.444419 | 7.85E-08 | 5.11E-07 | 6.946939 |
| IGKV1-16 | 1.027679 | 4.861938 | 5.433347 | 8.33E-08 | 5.41E-07 | 6.889891 |
| KRT6C | -1.03937 | 2.980199 | -5.41403 | 9.23E-08 | 5.95E-07 | 6.790596 |
| PI3 | -1.40446 | 5.864709 | -5.40188 | 9.84E-08 | 6.32E-07 | 6.728325 |
| IGHV3-23 | 1.001752 | 6.8119 | 5.396313 | 1.01E-07 | 6.50E-07 | 6.699829 |
| KRT15 | -1.14589 | 5.349449 | -5.34393 | 1.34E-07 | 8.43E-07 | 6.432994 |
| IGHV5-51 | 1.068299 | 6.799394 | 5.337954 | 1.38E-07 | 8.68E-07 | 6.402694 |
| IGKV1-9 | 1.016196 | 5.371824 | 5.31964 | 1.52E-07 | 9.50E-07 | 6.310052 |
| IGLV3-25 | 1.023994 | 6.6899 | 5.231869 | 2.39E-07 | 1.45E-06 | 5.870033 |
| IGKV4-1 | 1.067581 | 7.515438 | 5.192876 | 2.92E-07 | 1.75E-06 | 5.676676 |
| IGLV8-61 | 1.01854 | 4.207529 | 5.118932 | 4.26E-07 | 2.49E-06 | 5.313605 |
| IGLV3-21 | 1.015592 | 6.959306 | 4.934028 | 1.07E-06 | 5.89E-06 | 4.426484 |
| IGHV1-2 | 1.012847 | 5.271348 | 4.915532 | 1.17E-06 | 6.42E-06 | 4.339382 |
| KRT13 | -1.37253 | 5.04164 | -4.91243 | 1.19E-06 | 6.51E-06 | 4.324782 |
| SPRR2A | -1.32923 | 4.533719 | -4.84953 | 1.61E-06 | 8.66E-06 | 4.030998 |
| SPRR2E | -1.06357 | 3.121344 | -4.46499 | 9.72E-06 | 4.61E-05 | 2.310958 |
| SPRR2D | -1.06771 | 3.790193 | -4.45523 | 1.02E-05 | 4.80E-05 | 2.268976 |
| SPRR3 | -1.0656 | 3.811725 | -4.0231 | 6.55E-05 | 0.000272 | 0.497997 |
| NTS | -1.15885 | 4.209092 | -3.84849 | 0.000133 | 0.000523 | -0.16936 |
