# Supplementary material for: RGN as a prognostic biomarker with immune infiltration and ceRNA in lung squamous cell carcinoma
Source: Sci Rep. 2023 May 9;13:7553. doi: 10.1038/s41598-023-32217-z (PMC10170118; doi:10.1038/s41598-023-32217-z)
Supplement: Supplementary file 2 — Supplementary Table 2. [file 41598_2023_32217_MOESM2_ESM.docx]

Supplementary table 2

Correlation between RGN expression and related markers of immune cells in LUSC.

| Immune cell | Gene markers | Correlation score | P-value |
| --- | --- | --- | --- |
| B cell | CD79A | 0.320 | *** |
| CD8+ T cell | CD8A | 0.260 | *** |
|  | CD8B | 0.380 | *** |
| CD4+ T cell | CD4 | 0.430 | *** |
| M1 macrophage | NOS2 | 0.160 | *** |
|  | IRF5 | -0.0095 | 0.83 |
|  | PTGS2 | 0.220 | *** |
| M2 macrophage | CD163 | 0.390 | *** |
|  | VSIG4 | 0.400 | *** |
|  | MS4A4A | 0.410 | *** |
| Neutrophil | CEACAM8 | 0.250 | *** |
|  | ITGAM | 0.350 | *** |
|  | CCR7 | 0.320 | *** |
| Dendritic cell | HLA-DPB1 | 0.410 | *** |
|  | HLA-DQB1 | 0.320 | *** |
|  | HLA-DRA | 0.370 | *** |
|  | HLA-DPA1 | 0.380 | *** |
|  | CD1C | 0.370 | *** |
|  | NRP1 | 0.320 | *** |
|  | ITGAX | 0.340 | *** |

*P < 0.05, **P < 0.01, ***P <0 .001
